# The Role of Experimental Noise in a Hybrid Classical-Molecular Computer to Solve Combinatorial Optimization Problems

**DOI:** 10.1021/acscentsci.3c00515

**Published:** 2023-07-14

**Authors:** Veronica K. Krasecki, Abhishek Sharma, Andrew C. Cavell, Christopher Forman, Si Yue Guo, Evan Thomas Jensen, Mackinsey A. Smith, Rachel Czerwinski, Pascal Friederich, Riley J. Hickman, Nathan Gianneschi, Alán Aspuru-Guzik, Leroy Cronin, Randall H. Goldsmith

**Affiliations:** † Department of Chemistry, University of Wisconsin-Madison, Madison, Wisconsin 53706, United States; ‡ Department of Chemistry, University of Glasgow, Glasgow, G12 8QQ, United Kingdom; § Department of Chemistry, Northwestern University, Evanston, Illinois 60208, United States; ∥ Department of Chemistry, University of Toronto, Toronto, Ontario MS5 3H6, Canada

## Abstract

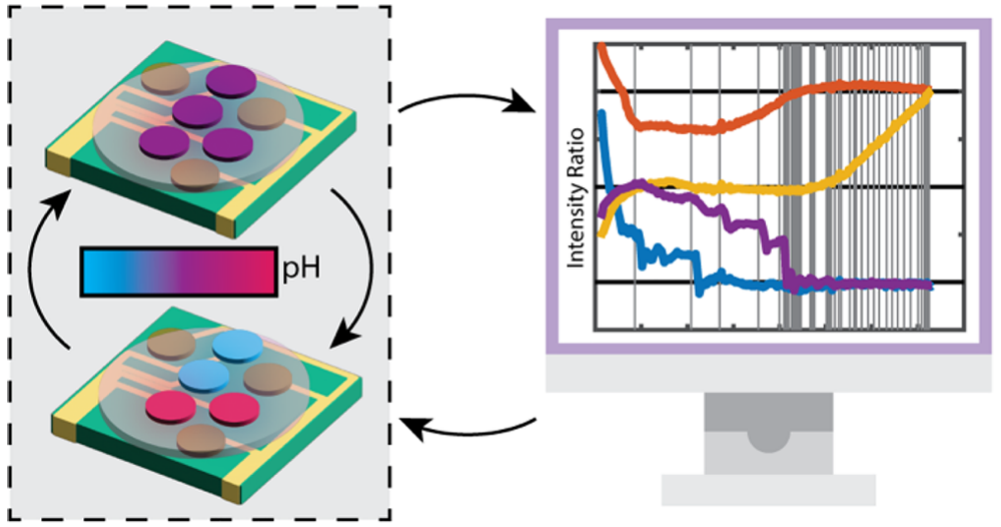

Chemical and molecular-based computers may be promising alternatives to modern silicon-based computers. In particular, hybrid systems, where tasks are split between a chemical medium and traditional silicon components, may provide access and demonstration of chemical advantages such as scalability, low power dissipation, and genuine randomness. This work describes the development of a hybrid classical-molecular computer (HCMC) featuring an electrochemical reaction on top of an array of discrete electrodes with a fluorescent readout. The chemical medium, optical readout, and electrode interface combined with a classical computer generate a feedback loop to solve several canonical optimization problems in computer science such as number partitioning and prime factorization. Importantly, the HCMC makes constructive use of experimental noise in the optical readout, a milestone for molecular systems, to solve these optimization problems, as opposed to *in silico* random number generation. Specifically, we show calculations stranded in local minima can consistently converge on a global minimum in the presence of experimental noise. Scalability of the hybrid computer is demonstrated by expanding the number of variables from 4 to 7, increasing the number of possible solutions by 1 order of magnitude. This work provides a stepping stone to fully molecular approaches to solving complex computational problems using chemistry.

## Abstract 2

A Hybrid Classical-Molecular Computer, featuring an array of electrochemical reactions with pH sensitive fluorescence signals as inputs and readouts, solves problems using inherent experimental noise.

## Introduction

The approaching limits of modern silicon computing motivate research into alternative computing paradigms. Silicon-based computers following a von Neumann architecture can be inefficient for some processes due to a limit on data throughput caused by the inherent separation of the memory and processing units. The number of transistors within a device has greatly increased, allowing for the execution of more complex tasks. However, high heat dissipation and power constraints,^1−3^ along with increasing costs and complexity in manufacturing, limit further increases of transistor density.^4^ Molecular or chemical-based computers may be one attractive family of alternative computing systems. Chemical computers have been developed based on reaction-diffusion systems and oscillating chemical reactions such as the Belousov–Zhabotinsky (BZ) reaction,^5−12^ while molecular computers have historically utilized DNA or other biological molecules to assist in computations.^13−17^ More broadly, molecular approaches to a variety of critical computational subsystems, including memory,^6,18−23^ image processing and recognition,^24,25^ digital circuits,^26^ and logic gates,^27−31^ are being pursued to investigate the nature of any perceived molecular advantage.

A hybrid classical-molecular computer (HCMC)^32^ couples chemical and digital analogues of a set of state variables. Having some tasks performed within a chemical medium and other tasks performed by traditional silicon components can allow certain advantages of molecular information processing to be accessed such as scalability, low power dissipation, and genuine randomness. Hybrid computing frameworks are designed with the intent to go beyond their individual computing components.^33−35^ For example, our HCMC can allow for programmability, which is a current limitation of purely chemical computing systems. Taken together, a demonstrative HCMC can provide substantial value as a stepping stone to evaluate molecular approaches to key computing subsystems, even though the approach is not fully molecular. Here, we present the implementation of an HCMC that consists of spatially distinct sites (we will refer to these “sites” throughout this paper) in an aqueous gel containing a payload of chemical reagents on top of a two-dimensional (2D) lattice of electrodes. To design and evaluate the HCMC, we have tested the hybrid computer on several well-studied problems in computer science, including problems that are representative of a nondeterministic polynomial time (NP)-complete class such as Boolean satisfiability problems, specifically 3-satisfiability (3-SAT), and number partitioning, as well as non-NP problems such as 2-satisfiability (2-SAT) and factorization. Importantly, we show that this architecture allows the chemical and electronic noise based in the physical infrastructure to directly and constructively influence complex algorithms in well-defined ways that may otherwise be expensive or suboptimal to achieve using a digital computer alone.

**Figure 1:**
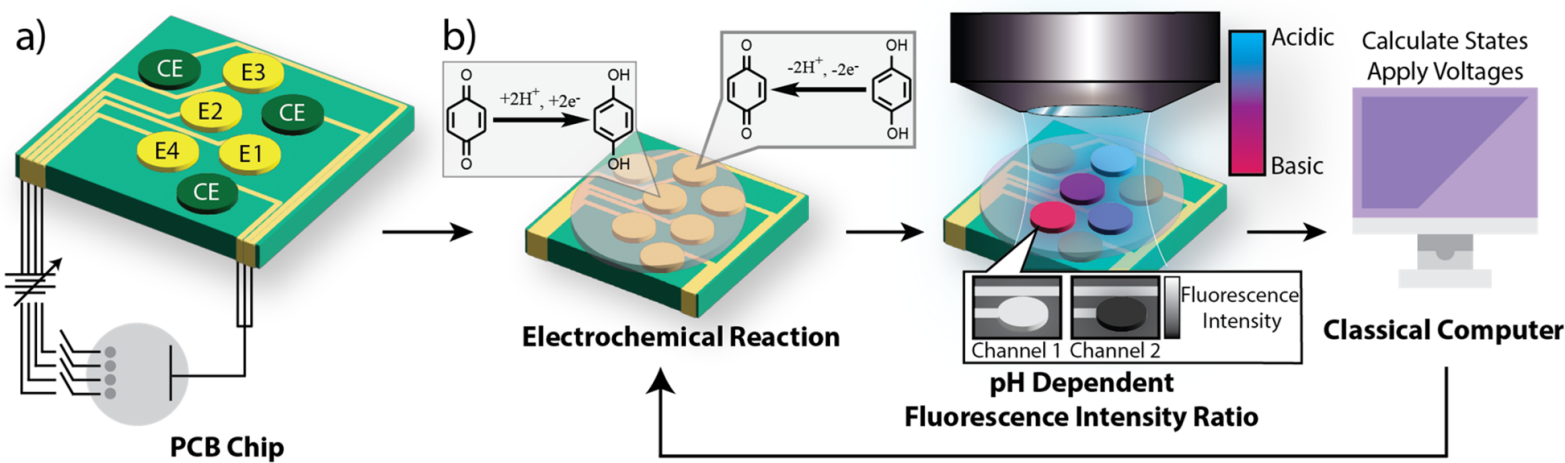
(a) Electronic schematic diagram of printed circuit board (PCB) chip featuring four individually addressable working electrodes (E1–4), and three counter electrodes (CE) connected to a programmable power supply. (b) General concept of hybrid classical-molecular computer (HCMC), electrode chip with electrochemical redox reaction of benzoquinone and hydroquinone resulting in changes to [H^+^] concentration. The pH changes are then measured optically using the fluorescence emission from a pH sensitive dye, which is spectrally separated to collect the ratio of the two emission peaks. The intensity ratio is then used in a classical computer to calculate the computational states of each variable and apply potentials at the electrode surface. This feedback loop is repeated until the computation converges.

The generalized HCMC architecture is shown in Figure 1 and consists of a simple feedback loop that couples information processing from a classical computer with a chemical system. This autonomous feedback loop forces the chemical and digital versions of a given variable to stay synchronous, enabling either physicochemical or digital events to dynamically update the information stored in both the digital and chemical registers, which are copies of each other. The chemical matrix, a gel, sits on top of a printed circuit board (PCB) chip with individually addressable electrodes that define the chemically active sites, Figure 1a. Importantly, the chemical variables encode information among the ensemble of molecules at the site proximal to each electrode. The chemical information comprising the states of the molecules over the working electrode sites is passed to the classical computer to manipulate the digital variables for processing, leading to output as potentials applied at the electrodes coupled to the chemical components, Figure 1b. In combination, the chemical and digital variables work together to solve computational problems, using properties unique to the digital and chemical environments to aid in processing.

For the chemical variables to be computationally useful, the chemical properties must be tunable in a manner that is reversible and responsive to electrochemical input signals and must themselves be capable of providing a robust signal that can be measured during readout. For this demonstrative HCMC, a simple redox reaction capable of bringing about reversible pH changes is employed, with a pH sensitive fluorophore, carboxy-SNARF-1, added to allow optical readout of the system states. The fluorescence readout is captured by cameras and is then processed by a classical computer, which converts the fluorescence reading into a digital state variable. The chemical and digital manifestations of each variable are interchangeable with a full two-way commuting relationship. Information is transferred from the chemical to the classical computer by fluorescence detection and from the classical computer to the chemical system by electrochemical control.

To use the chemical variables for problem solving, the HCMC must construct a many-to-one mapping between the microstates of the chemical system and an abstract mathematical formalism.^36^ For this purpose, we define the pH of the site to be analogous to the two spin states of an *idealized* Ising model^37^ or, equivalently, quadratic (two-local) unconstrained Boolean optimization (QUBO). The Ising model is a paradigm that can be used to solve hard combinatorial optimization problems, with a wide range of applications including logistical operation, biomolecule structural optimization,^38−40^ circuit design,^39,41^ and machine learning.^42,43^ Simulated annealing processors have previously been shown to be able to solve NP-Hard combinatorial optimization problems. Various types of digital and simulated annealers and Ising solvers have been developed,^44−51^ but our system uniquely utilizes a molecular fluorescent response both as an input to couple to the digital representation and as a readout of the final or interim states.

A given combinatorial optimization problem can be represented as a problem Hamiltonian, which then can be mapped onto the Ising Hamiltonian. A general Ising Hamiltonian for a two-state system of binary variables is defined as
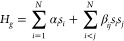
1where *s*_*i*_ and *s*_*j*_ represent spins (+1 or −1) for variables *i* and *j*, while α and β are problem specific coupling coefficients. The problem of choice is encoded into these coefficients, with α_*i*_ representing the local field for an individual variable as a vector and β_*ij*_ denoting the interaction energy or coupling between two variables as a matrix. To find the solution, one must find the optimal spin configuration such that the overall scalar value of *H*_*g*_ reaches a minimum given specific α and β.

The HCMC has two distinct overall inputs: the problem Hamiltonian that defines the external relationships between the sites as a function of the state that each site maintains and the initial values of the states, which can be any value between (−1, +1). The Hamiltonian matrix defines the sign and strength with which a pair of sites interact, represented by the β coupling coefficient. The interactions between the states along with the local field for the individual state, represented by the α term, are used in combination to yield a continuous scalar field over the space spanned by the state vectors. These couplings are a direct analogy to the mechanical linkages in a historical differential analyzer,^52,53^ which constrain the relationships between the variables of the mechanical analogue computer. The classical computer part of the HCMC also generates outputs used to induce couplings between the individual variables by actuating the electrodes. Specifically, the classical computer controls a potentiostat to apply voltages at the electrodes, inducing an electrochemical reaction and thereby changing the local pH. As the system evolves, the pH is measured and read out using a ratiometric fluorescent dye. The dye, carboxy-SNARF-1, allows for the ratio of its two fluorescence emission peaks to be linearly converted to pH, and in turn, converted to a numerical state value ranging from +1 to −1. By using a classical computer to host the information about site interactions virtually, interactions are not limited to nearest neighbors linked by diffusion and electrokinetic transport. Instead, the information contained in the sites can simply be linked by a classical computer, and the difficult engineering problem of creating tunable physical interconnects between sites is sidestepped. This compromise allows the HCMC to go beyond 2-body couplings, as defined by eq 1, and instead allows for high-order interactions based on the number of sites used. The Ising Model can then be redefined to describe interactions between higher-order terms with eq 2, where problems using *n* sites are defined by *h*^(*n*)^ tensors containing the *n*-site coupling terms.^32^
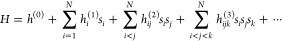
2This version allows for full connectivity between all of the variables encoded in the problem Hamiltonian, making higher-order coupling or interactions between sites possible. This arrangement expands the types of computational problems which can be tackled by this demonstrative HCMC.^32^

A gradient descent algorithm is applied in the classical computer to the computational representation of the state at each site so that the entire system moves toward a minimum or a solution to the computational problem. Gradient descent algorithms find minima by taking steps based on the steepness of the gradient with each step’s direction being dependent on the current state and the value of the function’s instantaneous gradient at that point. The step size is a scaling factor for how far the algorithm can move down the gradient at each step and is tuned by the user to optimize performance. For the HCMC, the position or gradient at each step uses the state value derived from the fluorescent output. The algorithm uses the slope of the scalar cost function for each problem, rather than calculating absolute values, which allows us to solve problems whose global minima have a nonzero absolute value. However, this approach is susceptible to converging to local minima.

The cost function for a problem can be rugged, with multiple, sometimes nearly degenerate, low energy local minima. For the HCMC to successfully solve the problem, it must identify and converge (halt) on the configuration corresponding to the lowest scalar value, the global minimum. Importantly, the HCMC can get trapped in local minima during the gradient descent and converge on the wrong solution. One common way to combat this process is to perform multiple initializations at different starting states to obtain a distribution of all converged states thereby increasing the probability of convergence toward the global minimum. Additionally, it has been shown that stochastic noise or random perturbations added to the optimizer, resulting in a stochastic gradient descent, can speed up the calculation by reducing the probability of being trapped in local minima.^51,54−56^ However, the way noise is generated is not always ideal. For example, all digital number generators are pseudorandom, meaning they can be predicted. An alternative can be found in the experimental measurements themselves. Intrinsic hardware noise has been reported as beneficial in solving combinatorial optimization problems using a memristor-based neural network system.^57^ Additionally, sensor noise in an optical cavity has also been theorized as a resource to increase sensitivity in low-power or noisy conditions.^58^ This phenomenon is true both for classical and quantum systems,^59^ but has not been explored in chemical systems.

By the design of computational paradigms that take advantage of the inherent experimental noise instead of deterring it, new chemical processes and designs can be used for chemical and molecular computing. The effect of noise on the computational efficacy inspired questions about how inherent experimental noise could aid or limit the annealing process in a HCMC.

## Results and Discussion

### Optical Experimental Setup

**Figure 2:**
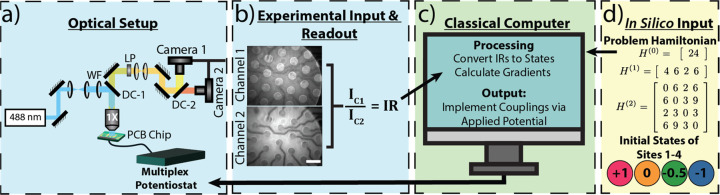
(a) Experimental optical setup for monitoring pH changes in chemical reaction gel on the 2D electrode array. (b) Fluorescence images of reaction gel used to calculate an intensity ratio (IR) which is used as an experimental input to the (c) classical computer to use in the gradient descent along with (d) the *in silico* inputs, such as the Ising Hamiltonian for the problem as well as the initial states for the sites at the first step of the computation. The classical computer outputs a potential at the electrode chip via a multiplexed potentiostat to induce pH changes monitored via the experimental readout, IR. (2 mm scale bar, WF – Widefield lens, DC-1-505 nm dichroic mirror, DC-2-610 nm dichroic mirror, 1× Objective, LP – 532 nm and 540 nm long pass filters).

The HCMC includes a purpose-built microscope, Figure 2a, with wide-field illumination provided by a 488 nm laser. Fluorescence is collected using a 1X air objective and directed toward cameras, passing through a 505 nm long pass dichroic mirror, a 532 nm long pass filter, and a 540 nm long pass filter to remove excitation light. The fluorescence is spectrally resolved into two color channels using a 610 nm long pass dichroic mirror and focused onto two separate cameras which capture the emission from the two fluorescence peaks of the pH indicator.

### Electrode Array Design

We use a simple and cost-effective custom-made electrode array with an electrode size of 1 mm placed in a hexagonal grid to maximize the connectivity of multiple working electrodes with counter electrodes. All electrodes were gold-plated by the PCB manufacturer. The electrode arrays were fabricated using standard PCB manufacturing; see Supporting Information (SI) for details.

### Chemical Encoding, Input, and Readout

The chemical system for encoding information in the HCMC is a hydroquinone/benzoquinone redox couple dissolved in an aqueous buffered F-127 Pluronic gel containing a fluorescent reporter. The gel is pipetted onto a PCB-based electrode chip.^60^ The Pluronic gel allows for a solidlike matrix reducing diffusion across the electrode chip, which keeps the chemical changes localized over the specific electrode surface but does not hinder electrophoretic motion. The quinone redox couple allows for a reversible way to manipulate pH when it is paired with applied potential from the electrodes. There is around a 1 pH difference between the −1 state and the +1 state for each site participating in the computation. The formulations and specific pH values for each site corresponding to the computational states are described in the SI.

Readout is provided by fluorescence, which optically assesses the pH of the gel region over each electrode and thereby conveys the computational state of each variable. These fluorescent measurements allow for a real-time, in situ readout.^61^ To optically monitor pH, we used a ratiometric pH-sensitive fluorophore with a pH-dependent emission spectrum,^62,63^ Carboxy-SNARF-1, which has previously been used to measured intracellular pH.^64−68^ The fluorescence emission has two peaks at 580 and 640 nm, and the ratio between these two peaks can be calibrated to read out pH. The use of a ratiometric pH sensor is critical, as a pure intensity readout can be changed by interference from electric field-induced concentration fluctuations, aggregation, and photobleaching. The intensity ratio (IR) between these two channels, which is proportional to pH, is passed to the classical computer and converted into a state value, Figure 2b,c. The fluorescence signal is therefore used not only as a readout of the chemical information but also as an input to the classical computer.

### Classical Processing and Output

**Figure 3:**
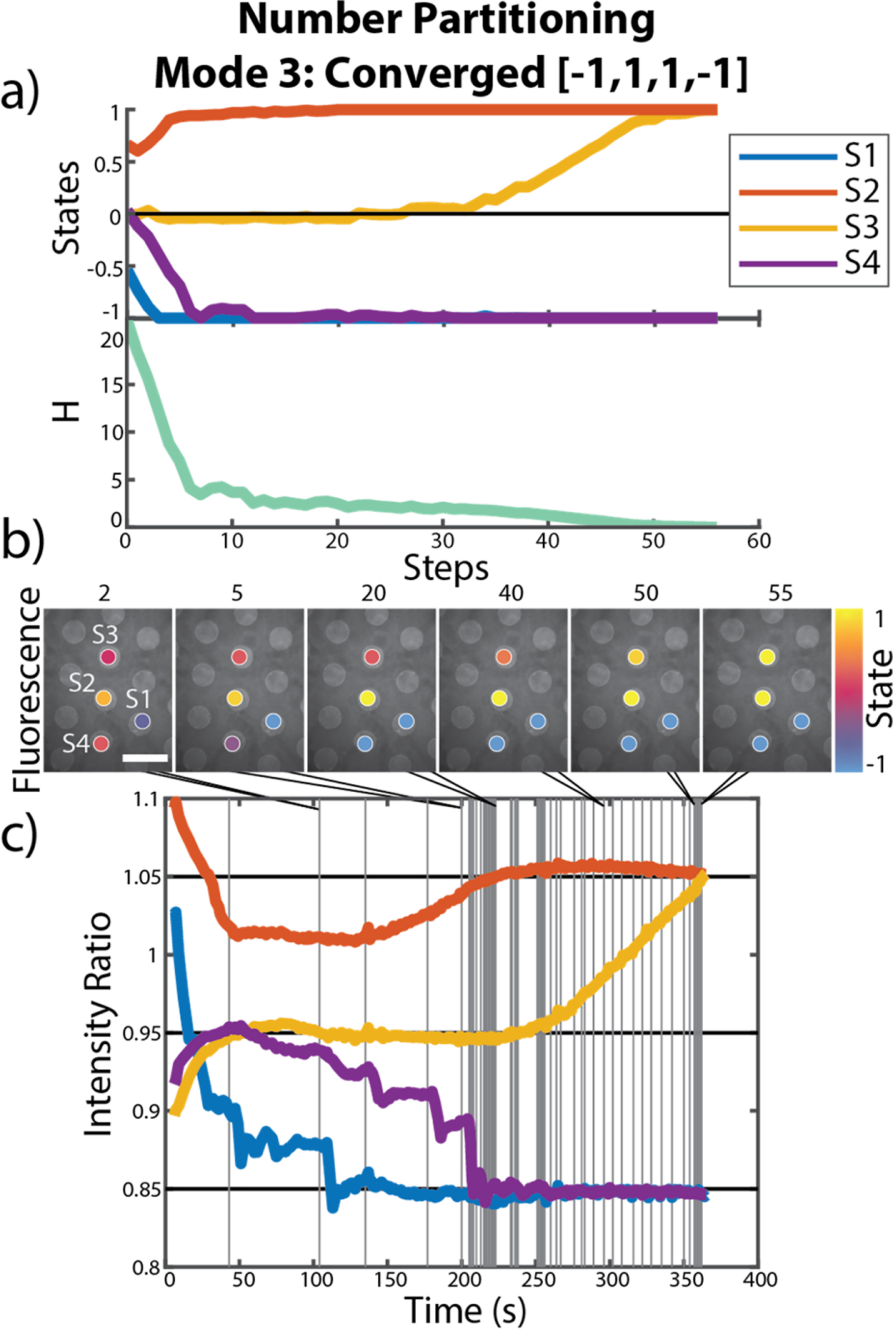
Progression of the hybrid classical-chemical computer solving a Number Partitioning problem using Mode 3 (see the text for details). (a) Evolution of states throughout the computation, where S1–S4 represent the four sites, and the value of the problem Hamiltonian (H) at each step of the computation. (b) Fluorescence images of the reaction gel on the electrode chip with artificially colored circles depicting the state value at various steps, scale bar is 2 mm. (c) The value of the intensity ratios over time during the computation, where vertical lines represent each step of the computation.

To process the ratiometric responses from these images, the coarse location and approximate size of electrodes participating in the computation are initially determined manually and refined using image segmentation that employs a watershed algorithm^69^ to identify the electrode region, Figure 3b, all before the computation begins. The state values, converted from the experimental IRs, are used as the input in the gradient descent calculation performed by the classical computer, Figure 3. As previously mentioned, the gradient descent algorithm uses the state values along with the user-determined step size to determine the target state values for the next step. Alternatively, a stochastic gradient descent algorithm can be utilized through the addition of random computer-generated noise to the target states. This noise will be termed *in silico* noise and can be optionally added. To achieve the determined target state, a potential is applied at the electrodes via a multiplexed potentiostat, which allows independent iterative control of up to 7 electrodes, Figure 2b. A proportional-integral-derivative (PID) algorithm is used to reach the target IR corresponding to the target state with the PID gains tuned to avoid overshooting by the potentiostat. The PID loop consists of the potentiostat manipulating the potentials over the electrodes while the changing IRs are monitored using the optical setup, Figure 3c. Once the IR values at all participating sites are within a set threshold of the target, the PID loop for that step in the gradient descent is finished, and the next step can be taken. This is illustrated by the vertical lines shown in Figure 3c. The user-set threshold determines how close the experimental IR needs to be to the targeted set point before proceeding to the next step. This PID loop is performed for each step of the gradient until the minimum is found. A movie of the fluorescence response is provided in the SI, showing the HCMC using 7 sites (working electrodes) where the PID gains were set to induce an exaggerated fluorescence response. Combining all of these parts, the HCMC can be successfully run starting at either random or specific initial states and converging on the global minima, thus solving various optimization problems, including number partitioning, Figure 3, and 2-SAT, Figure S7.

An accompanying numerical simulation engine was developed describing the various physical phenomena that occur during the computation when using the HCMC. The simulation engine creates a generalized electrode array network by combining Kirchhoff’s equations coupled with a Secondary current distribution model. The calculated time-dependent current profiles are then combined with a buffer dynamics model to describe localized pH changes over the electrodes (see SI). Combined with a dynamic pH control algorithm using proportional logic, this supporting simulation demonstrates good experimental resemblance, as well as explains the hallmarks of experimental non-idealities, such as ringing in the experimental PID loops, and can aid in parameter optimization as well as instantiating a large-scale problem on an electronic chip.

### Evaluating the Role of Experimental Noise

The HCMC is designed to solve quadratic combinatorial optimization problems using a gradient descent algorithm with a fluorescent molecular signal as the input and readout. As previously mentioned, added stochastic noise aids convergence to a global minimum by preventing the possibility of getting stuck in local minima, saddle points, or plateaus. Importantly, we examined the impact of HCMC’s intrinsic noise when solving computations.

Three operational modes of the HCMC were investigated: Mode 1 uses what have been termed “idealized states”, where the states in each step of the gradient are identical with the set points determined by the classical computer; Mode 2 uses “*in silico* states”, which are idealized states but with an added stochastic *in silico* noise component, resulting in a stochastic gradient descent; Mode 3 uses “measured states”, which convert the experimentally achieved IR signal into the state for the computation with no added *in silico* noise. As Mode 1 does not include any noise component, the HCMC is expected to either not be able to progress through the computation, in contrast to the run displayed in Figure 3, or be more likely to get trapped in local minima, resulting in convergence on incorrect answers. Mode 3 also does not include an *in silico* noise component; however, the experimental noise within the measurement is expected to be a beneficial source of stochasticity with potentially large enough fluctuations to avoid repeating the failed trajectories observed in Mode 1.

**Figure 4:**
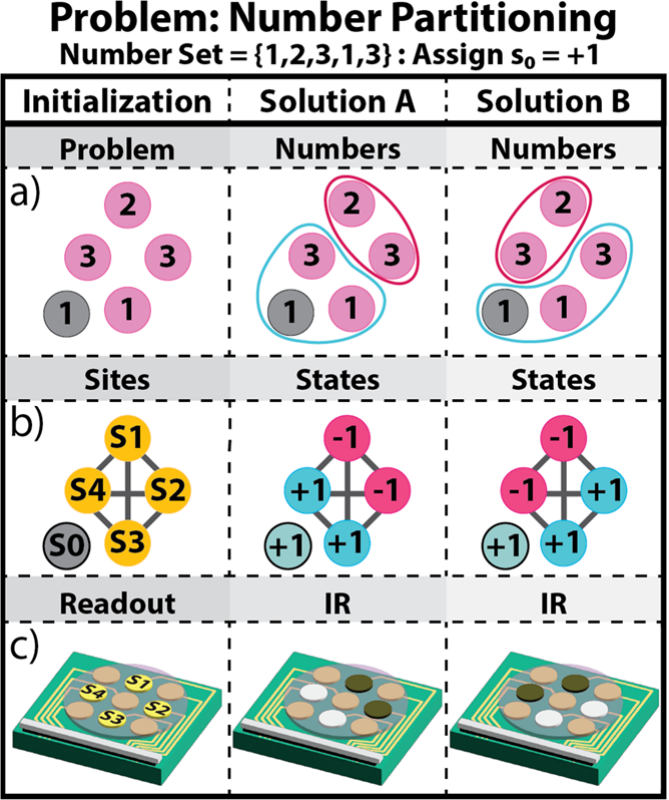
Visual depiction of the number partitioning problem and two correct solutions. The three columns correspond to the numbers in the problem (left), the grouping for solution A (center), and solution B (right). (a) The problem represented by the numbers in the problem, (b) the state values at Sites S1–S4, and (c) depiction of changes observed on the electrode chip via fluorescence.

To investigate the role of experimental noise, multiple complementary experiments involving number partitioning problems were performed. Number partitioning is a NP-complete problem, which asks: for a given set of numbers, how can they be divided into disjointed subsets with equal sums? Number partitioning has been called the “easiest hard problem”^70^ in terms of its complexity. For the purpose of investigating the role of noise, we have selected a simple problem consisting of a small number of variables that still produced a nontrivial cost function with multiple solutions. The number set {1,2,3,1,3} was selected, and an Ising Hamiltonian for the problem was generated, Figure 2d; see SI for details regarding Hamiltonian generation. The Hamiltonian corresponding to this problem is non-negative and minimized at *H* = 0, when the sums of the two sets are equal. This five-number set was expressed using four variables with the first number {1} automatically assigned to a +1 state (*s*_0_) without a loss of generality. The other four numbers are assigned to the spatially distinct gel areas over individual electrodes, referred to as sites. The state values (*s*_*i*_) for the remaining numbers {2,3,1,3} describe to which subset each number belongs and will be defined by the fluorescence IR values at Sites 1, 2, 3, and 4. A depiction of this problem can be seen in Figure 4. The correct partition solution (*H* = 0) for this problem is {1,1,3} and {2,3}. However, as the number three is repeated twice in the set for our problem, there are two different ways this partition can be expressed using our sites. One solution, Solution A, is where the number two associated with Site 1 would be paired with the number three associated with Site 2, Figure 4a – Solution A. The second solution, Solution B, has the number two associated with Site 1 paired with the number three on Site 4 instead, Figure 4a – Solution B. To express that these numbers are grouped together, the state values of these sites must be the same, Figure 4b.

Starting with Solution A, the state values for Sites 1 and 2 should be the same, meaning that both sites should either be +1 or −1. However, there is an additional constraint: the first number in the number set is already assigned a +1 state. Consequently, Sites 1 and 2 are unable to take the +1 state, as this would result in the partitioning of {1,2,3} and {1,3}, which is incorrect. Therefore, Site 1 and Site 2 must take a −1 state, which gives Solution A [−1,–1,1,1], visually depicted in Figure 4 – Solution A. For the second solution, Site 1 is paired with Site 4, both will take the −1 state, resulting in Solution B [−1,1,1,–1], Figure 4 – Solution B.

**Figure 5:**
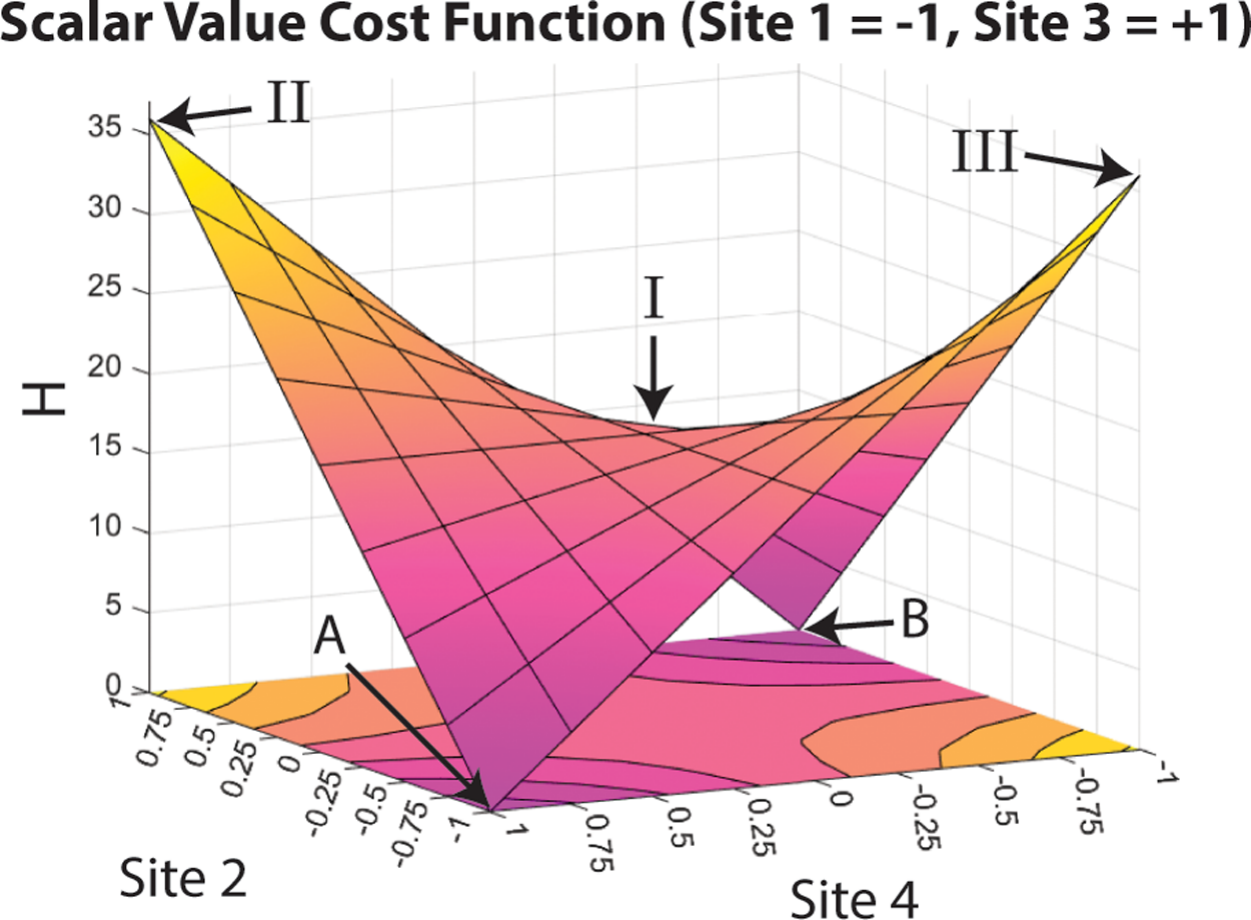
Scalar value cost function for the Number Partition Hamiltonian when Sites 1 and 3 are kept constant (at −1 and +1, respectively) and Sites 2 and 4 are varied from −1 to +1 states. Labeled states correspond to (I) Saddle point [−1,0,1,0], (II) first maximum point [−1,1,1,1], (III) second maximum point [−1,–1,1,–1], and Solution (A) [−1,–1,1,1] and Solution (B) [−1,1,1,–1].

Interestingly, Solutions A and B differ by two variables, the second and fourth, both of which have oppositely signed values of 1. When plotting the cost function for this problem with respect to Sites 2 and 4, and with Sites 1 and 3 at constant solution state values (−1 and +1 respectively), a symmetric saddle shape emerges, Figure 5. The flat saddle point is located at [−1,0,1,0], Figure 5-I. The value 0 here represents the midpoint state value between the two extreme state values, −1 and +1. There are two maxima located in Figure 5-II at states [−1,1,1,1], and in Figure 5-III at states [−1,–1,1,–1], with equal values for the problem Hamiltonian. The landscape also shows the two solutions, Figure 5-A [−1,–1,1,1] and Figure 5-B [−1,1,1,–1], both with the problem Hamiltonian equal to 0. Based on this function, three separate initial states were selected as starting points to explore how noise affects system evolution: [−1,0,1,0] (Initial State I, Figure 5-I), [−1,1,1,1] (Initial State II, Figure 5-II), and [−1,–1,1,–1] (Initial State III, Figure 5-III).

**Figure 6:**
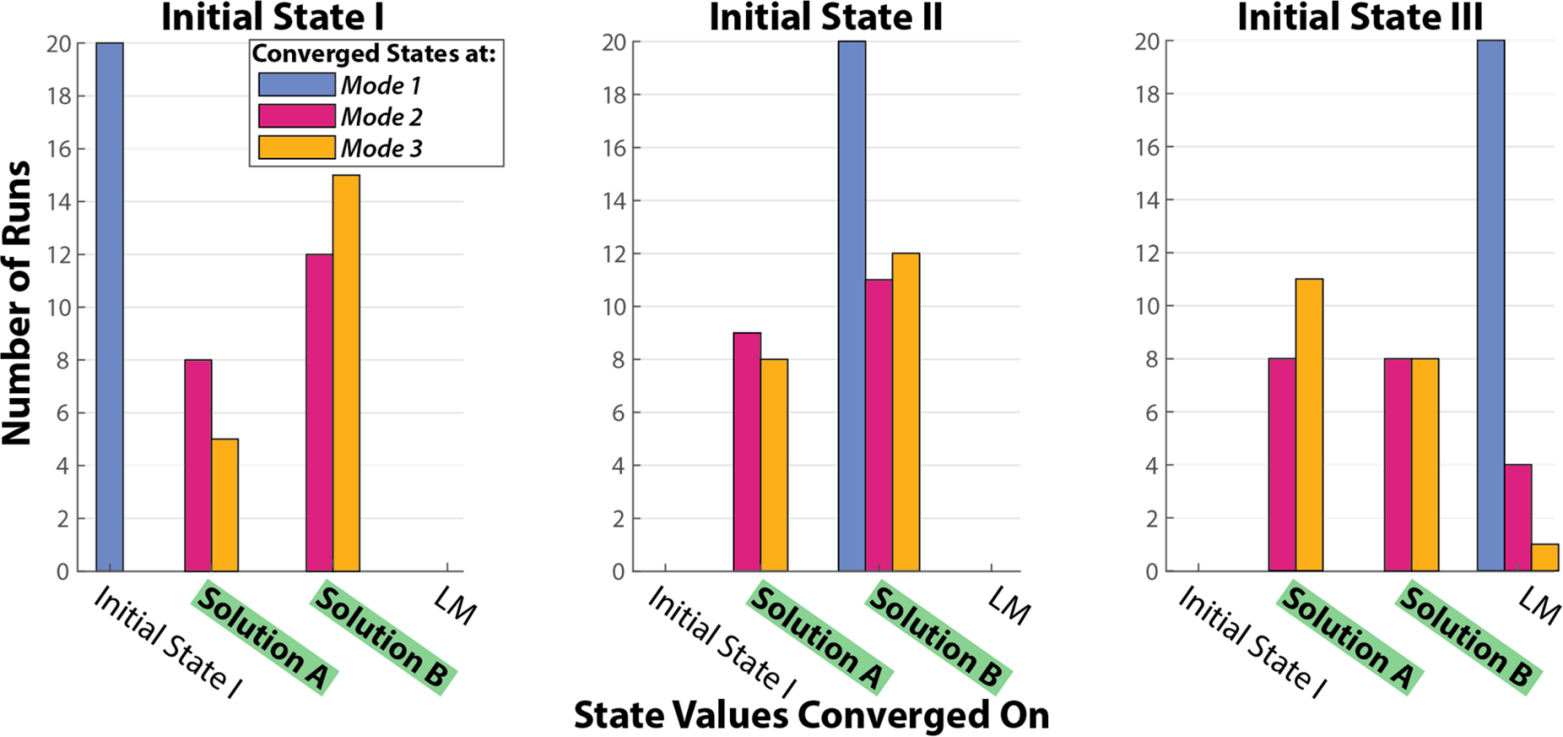
Bar graphs showing the distribution of answers, where each graph corresponds to a separate initial state, with Initial State I (left), Initial State II (center), and Initial State III (right). The states converged by our system correspond to the Initial State I, the two solutions, A and B, and a local minimum (LM). Colors correspond to the three different operational modes for the HCMC.

For these experiments, the HCMC was run using each of the 3 operational modes described above, Mode 1 using idealized states, Mode 2 using *in silico* states, and Mode 3 using measured states. Twenty repeats were performed for each mode at each of the three initial states, resulting in a data set of 180 runs; see Table 1. The distribution of the converged states is shown in Figure 6.

**Table 1:** Distribution of HCMC-Found Solutions of Number Partitioning Computations

	Mode 1 – Idealized States	Mode 2 – *In Silico* States	Mode 3 – Measured States
Initial State I	A: 0/20	A: 8/20	A: 5/20
	B: 0/20	B: 12/20	B: 15/20
	Initial State I: 20/20		
Initial State II	A: 0/20	A: 9/20	A: 8/20
	B: 20/20	B: 11/20	B: 12/20
Initial State III	A: 0/20	A: 8/20	A: 11/20
	B: 0/20	B: 8/20	B: 8/20
	LM: 20/20	LM: 4/20	LM: 1/20

When starting at the saddle point (Figure 5-I) and using Mode 1, the idealized states, the computation immediately and erroneously converges on the initial states [−1,0,1,0] for all the runs carried out in this mode (100%), Figure 6. Here, with the starting point at the saddle and without the addition of any stochastic *in silico* noise, the gradient experiences a flat slope and incorrectly determines this scenario to be a minimum, as expected. When repeating the computation starting from the same initial state but with Mode 2, *in silico* states with noise, the computation was always able to converge on one of the two correct solutions, with a 40:60 split between Solutions A and B. Excitingly, with Mode 3, which uses the experimentally determined measured states, the computation is once again able to run smoothly and converge to both global solutions, resulting in a 25:75 split between Solution A and B across all runs, a similar distribution of converged states as when using Mode 2. This result is important, as it clearly shows that the experimental noise inherent to the empirical measurement in the chemical system is significant enough to allow the HCMC to leave this flat portion of the energy landscape while not impeding convergence.

We then switched to an alternate starting point: one of the maxima on the cost function Figure 5-II. Without any additional noise at Mode 1, the HCMC is only able to find one of the two correct solutions (100%). In contrast, runs at both Modes 2 and 3 were able to converge on both solutions with similar distributions; see Table 1.

**Figure 7:**
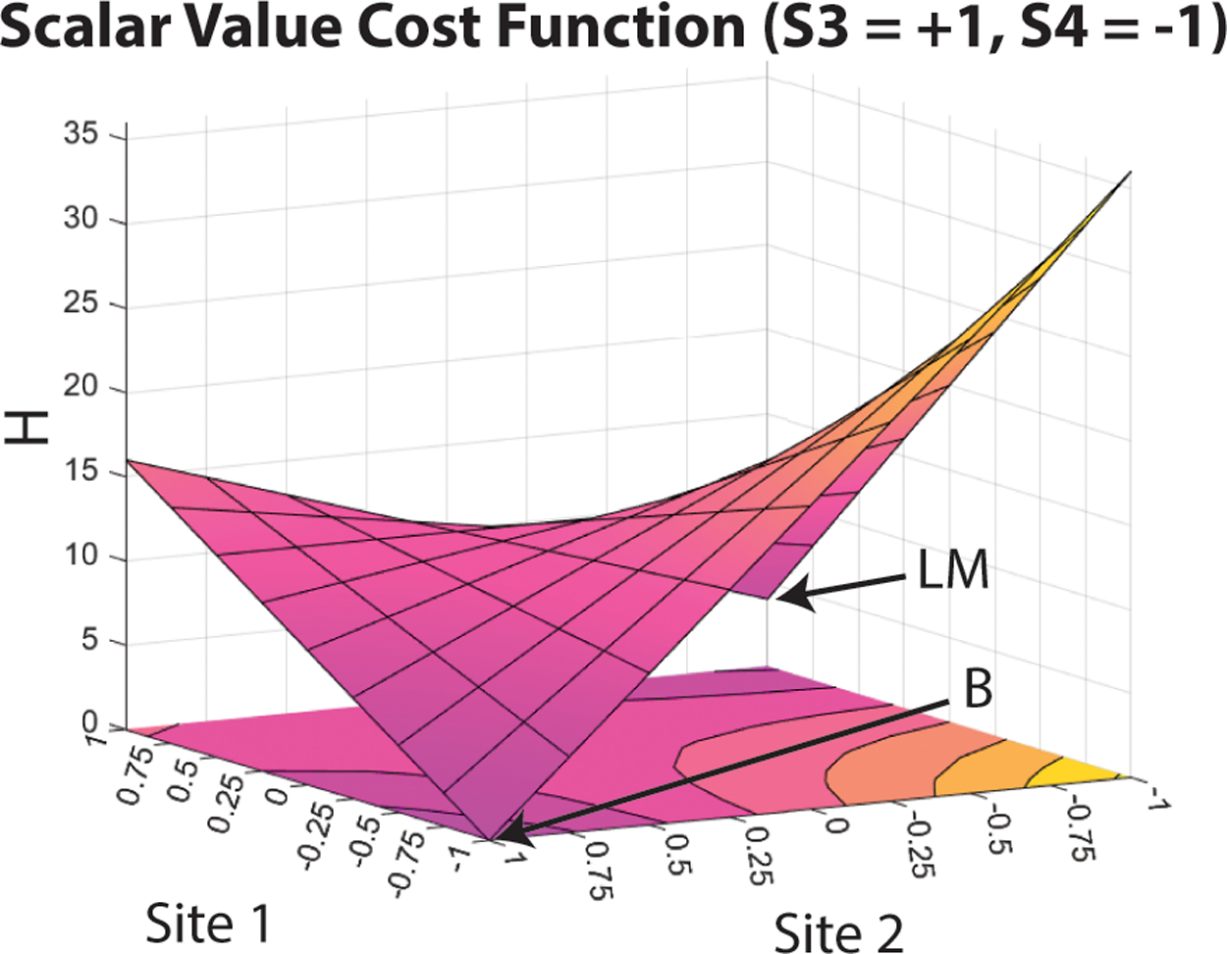
Scalar cost function for Number Partitioning Hamiltonian where Sites 3 and 4 are kept constant at +1 and −1, respectively, and Sites 1 and 2 are varied from +1 to −1. Two minima are observed, (B) a global minimum at state values [−1,1,1,–1] corresponding to Solution B, and (LM) a local minimum at state values [1,–1,1,–1].

Finally, when starting at the second maximum, Figure 5-III, runs at Mode 1 all converged on states [1,–1,1,–1] (100%), which does not match either of the two solutions or the initial state. For this set of converged states, the state value for the first site has flipped, from −1 to +1. When plotting the cost function for this case, it is illustrative to switch to Site 1 and Site 2 as the independent variables, Figure 7. We see another saddle, but this time it is asymmetric, with the global minimum at one of the correct solutions, Solution B, Figure 7-B and a new local minimum (LM) at states [1,–1,1,–1], Figure 7-LM. When using Mode 2 we see a split of 40:40:20 between solutions A, B, and the LM. Excitingly, runs at Mode 3 also demonstrate a distribution of the converged states, with a 55:40:5 split between Solutions A, B, and LM. This again supports that the intrinsic experimental noise is beneficial to the HCMC to solve computations, seen previously with experiments starting at Initial State I. Additionally, the magnitude of the noise present also shows a benefit in reducing convergence at local minima, with similar statistical results compared to Mode 2, using the *in silico* states. Therefore, it is reasonable to run the HCMC using the measured states with experimental noise in lieu of *in silico* noise.

### Sources and Magnitude of Experimental Noise

While the above results show how experimental noise benefits the function of HCMC, there are still unanswered questions about the origins and magnitude of the experimental noise. As the complexity of computational problems changes, the amount of noise that is beneficial vs inhibitive changes as well. By identifying the sources of experimental noise within the HCMC, it becomes possible to program or tune the noise, depending on the complexity of the problem. As mentioned earlier, the optimal noise to carry out a stochastic process has been heavily studied in the context of classical and quantum dynamics.^59^

For the experimental noise to impact the computational ability of the computer, it must be present in the experimental input used in gradient descent. The experimental input originates from the measured fluorescence-detected IRs, which are based on the electrochemically generated pH changes. Therefore, the magnitude of fluctuations in the optical measurements of fluorescence or the electrochemical components responsible for the change in pH must be determined. As these fluctuations will be quantified in terms of intensity ratios, it is possible to convert the noise to state variables and obtain the relative scale of the noise in state space. In other words, the conversion allows us to compare the experimental noise to the *in silico* noise added in the previous section when running the HCMC at Mode 2.

Before investigating the experimental noise, it is necessary to discuss what exactly qualifies as *in silico* noise. The *in silico* noise in the HCMC is a randomly selected number from a normal Gaussian distribution generated via Python. The Gaussian distribution is centered over the integer zero with the standard deviation or width of the distribution specified by the user. The randomly selected number is applied to the state value after the next step in the gradient is calculated but before it is executed. As the offset applied at each step changes, the standard deviation selected by the user is what will be broadly referred to as the *in silico* noise. For the experiments above, runs at Mode 1 had a standard deviation of 0 selected, while at Mode 2 a standard deviation of 0.1 was selected.

Runs at Mode 3, using measured states, had a standard deviation of 0, meaning no *in silico* noise was added. Importantly, however, there is experimental noise present that allows the HCMC to perform successfully. To determine the magnitude of experimental noise, the noise in the optical measurements was first quantified. A reaction gel containing SNARF-1 dye was examined under the same conditions used in the computational runs. The reaction gel was imaged for 5 min without an applied potential, and the standard deviation in the average fluorescence IR signal over the electrodes was quantified. This measurement includes noise from the imaging setup, such as the read noise of the cameras and the noise in the intensity of the laser, as well as any background fluorescence from the electrode chip. The standard deviation was converted into an effective *in silico* noise value by using the linear relationship between the IR and state. The percent standard deviation along with the calculated equivalent *in silico* noise value are shown in Table 2. The equivalent *in silico* noise value calculated is an order of magnitude smaller than what was used in Mode 2.

**Table 2:** Quantified Experimental Noise

Description	Percent Standard Deviation (%)	Calculated Relative *In Silico* Noise
Noise from Optical Measurement	0.22 (±0.05)	0.02
Noise from Electrochemical Measurement	0.0010 (±0.0002)	0.0001
Noise from Complete Experimental Measurement	0.25 (±0.05)	0.02

For the electrochemical apparatus, the noise in the current at each electrode was measured under a variety of conditions. This measurement includes noise in the potential applied on the electrode surface, fluctuations in current caused by interactions at the electrode surface, and noise in the measurement of the current itself. The current was measured by the potentiostat while performing a controlled potential chronoamperometry experiment. A gel was placed onto the electrode chip and imaged in the same way as described above but with various constant applied potentials. The potential in each case was held constant for 5 min while the current was measured. The noise in applied potential alone was also measured independently but was found to be insignificant; see SI. The measured current traces were converted to charge traces, which were used to determine the gain or loss of protons over time due to the oxidation or reduction of the quinone couple. By assuming 100% Faradaic efficiency, a maximum possible contribution from current noise can be determined as each fluctuation in the measured current is assumed to reflect changes in the production or loss of protons in solution at the electrode. After accounting for buffering, these fluctuations in proton concentration (and thus pH) can be equated to a change in the IR over time. As shown in Table 2, the maximum contribution to the experimental noise from the electrochemical apparatus is significantly less than the contribution from the optical measurement.

Finally, to investigate how the optical and electrochemical components of the noise collectively contribute to the noise in the IR, a gel was imaged while maintaining a set IR over many minutes. The gel was initiated at pH 7 and then potentials were applied using the PID loop to bring all the active electrodes to approximately pH 7.5 (state = −1, IR = 0.85). This experiment combines the noise contributions from the two previous experiments discussed above. The noise calculated from these experiments was comparable to the optical noise measurements, Table 2.

From these experiments, the noise in the optical measurements appears to be the biggest contributor to the overall experimental noise with the largest standard deviations. To understand what effect this magnitude of noise has on the computational solving ability of the HCMC, we return to the previous number partitioning problem. When starting at Initial State III, convergence at a local minimum was observed. At Mode 1 with no *in silico* noise, the HCMC always converged on the local minimum. Switching to Mode 2, with an *in silico* noise value of 0.1, the frequency of local minimum convergence drops to only 20%. The computation was repeated at Mode 2 starting at Initial State III at varying *in silico* noise values (see SI). The HCMC needed at least an *in silico* noise value of 0.01 to find the global minima; however, the success rate was fairly low, with only 26% of runs converging correctly on global minima and 74% converging on the local minimum. Increasing the *in silico* noise to 0.02, a magnitude similar to the experimental noise, the HCMC performance improves to 54% convergence at global minima. This supports that the experimental noise derived from the optical measurement is significant enough to benefit the HCMC’s performance.

To establish the contribution from fluctuations in the molecular population above the electrode surfaces, the Poisson noise in the SNARF-1 population was estimated.^71^ The number of SNARF-1 molecules present in the gel over a single electrode surface in the implemented HCMC is around 5 × 10^13^ molecules. With this number of molecules, the relative fluctuation in the number of SNARF-1 molecules in a particular protonation state would be around 1 × 10^–7^, which is too low to impact the trajectory of the HCMC when starting at Initial State III. This estimation further supports that the major contributor to the experimental noise in this implementation of the HCMC is from the optical measurement itself.

### Seven Electrode Computations and Beyond

**Figure 8:**
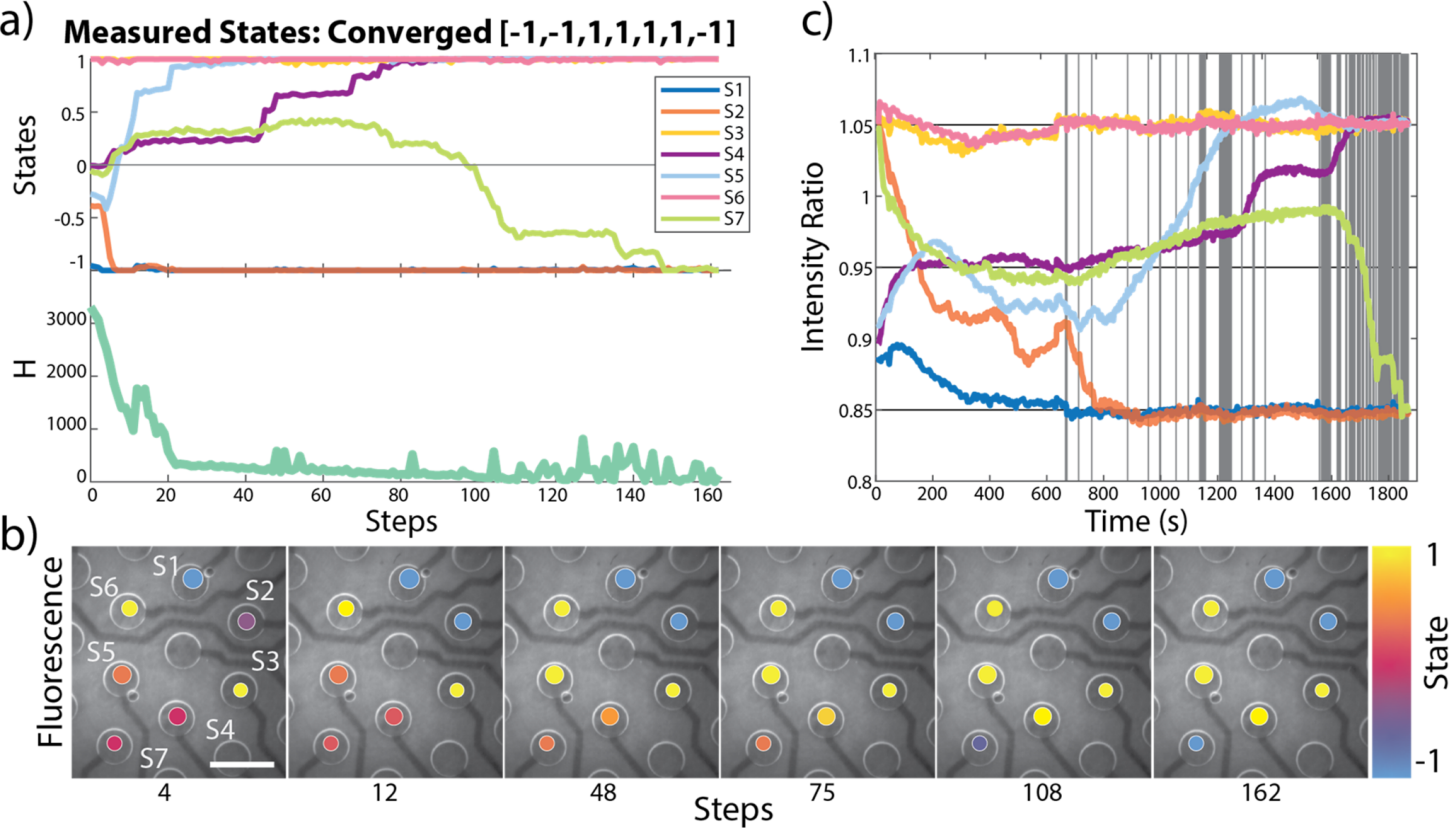
Progression of a computation by the hybrid classical-chemical computer solving a prime factorization problem with seven working electrodes. (a) Evolution of states throughout the computation, where S1–S7 represent the seven sites, and the value of the problem Hamiltonian (H) at each step. (b) Fluorescence images of the reaction gel on the electrode chip with artificially colored circles depicting the state value at various steps; scale bar is 2 mm. (c) The value of the intensity ratios over time during the computation, where vertical lines represent each step in the computation.

A benefit of the HCMC platform is the ability to increase the number of variables inexpensively. Scaling up the number of electrodes used allows the HCMC to tackle higher variable counts and more difficult computational problems. To explore this ability, we increased the number of working electrodes to seven to access new computational capabilities. For this implementation of the HCMC, there was a limit of seven working electrodes based on the specific multiplexed potentiostat used. The HCMC at Mode 3, that is, using measured states as the input, was used to solve 3-SAT problems with 7 variables with 28 clauses (shown in Figure S8). The 3-SAT problem is an NP-complete problem that asks whether a set of clauses in propositional logic is satisfiable. 3-SAT specifies that there are at most 3 variables within each clause. The HCMC has also successfully solved number factorization problems, as demonstrated by the decomposition of 91 into prime factors 7 and 13 (Figure 8). Details about the generation of the problem Hamiltonian can be found in SI. Prime factorization is a problem in the computational class NP, where given an integer (*N*) the goal is to find the two prime numbers whose product is *N*. This problem has two solutions, 7 × 13 and 13 × 7, which are expressed in binary numbers by the states. We ran this factorization problem to the point of convergence on the HCMC 10 times, with 8 of the 10 runs resulting in convergence on the correct solution.

To continue to solve more complex and higher value problems consisting of more variables, more sites, and therefore, electrodes, will be needed, and a discussion of the scaling of variables and need for distinct sites can be found in the theoretical simulations performed by Guo et al.^32^ Scaling up to increased variable counts beyond those in the current work could be achieved straightforwardly by using larger numbers of electrodes addressed within a larger optical field of view. The use of microelectrode arrays would allow for hundreds or thousands of electrodes to fit in an area even smaller than the active area in this work,^72^ while increased magnification and camera pixel arrays would enable the corresponding increase in readout, allowing for increased complexity and computational power in the same form factor through miniaturization. While physically increasing the number of electrodes would be relatively simple through alternative chip design, the actual implementation of accessing hundreds (or thousands) of individually addressable electrodes, along with maintaining and changing the pH over those sites, would be nontrivial. The ability to control via a potentiostat each electrode could be challenging to scale, while adjustments to the experimental PID controls would be required to ensure stable pH changes and minimize electrochemical side reactions at each site. One way to handle this scaling is to use microdroplets with programmable payloads to optimize the behavior of the electrode–microdroplet pair, where compartmentalization can enable enhanced local programmability and, consequently, performance.^32^ Finally, fine-tuning the computational parameters would be required to access the full scalability benefits of the HCMC. Still, none of these problems are intractable, and there are additional benefits that could be achieved through miniaturization, as discussed below. One hundred electrodes would be sufficient to capture a Traveling Salesperson optimization with ten variables.^32^

In the limit of very small microdroplets of solution on these arrays, the HCMC could even retain high readout signal-to-noise while benefiting from Poisson noise among the now small molecular population as a new source of stochasticity, as discussed above. In this regime, the Poisson noise, which is white and truly random, could be easily controlled by modulating the size and concentration of the microdroplets.^73^ To achieve an HCMC state standard deviation of at least 0.02 from Poisson fluctuations of emissive molecules, a population of at most 2500 molecules would be needed, easily achievable, and visible within microdroplets.

This stepping stone has also identified a number of challenges of this specific approach as well as chemical approaches in general to achieve a fully chemical computer that can compete with classical and even quantum implementations. Issues with clock speed will be ever present in systems requiring significant mass transport, though miniaturization can help to reduce this gap. Solution-phase molecules, with chemically identical environments, should allow for reproducible dynamics, but this reproducibility has not yet been demonstrated in chemical computing and should not be taken for granted, particularly in mesoscale implementations. Stability in certain chemical systems has been demonstrated to be extremely high,^19,22^ but the need for optical readout and electrochemical cycling in our system, and the degradation processes that result, will certainly require additional optimization and may constitute important technology hurdles. Still, in some cases the need for inexpensive implementations may allow some degree of toleration of slower speeds or decrease component lifetime.^32^

## Conclusion

We have designed a programmable hybrid classical–molecular computer that maintains a set of state variables encoded both digitally and chemically. Digital information is stored conventionally *in silico*, while the chemical information is encoded in a pH-sensitive gel on top of an electrode array. Changes to the state variables can be communicated via a feedback loop between the digital and the chemical variables. Spectroscopic monitoring of pH using a ratiometric dye transfers information from the chemical domain to the digital domain. Information then transfers from the digital to the chemical domain via electrochemical potentials applied by an electrode array. Such an architecture enables chemical and digital operations in either domain to concurrently modify the state variables, enabling the execution of a single algorithm distributed across the two physical domains. The role of the intrinsic experimental noise within the HCMC was investigated and shown to be beneficial to solve classic NP-hard problems, without the need for *in silico* noise (pseudo random numbers) which is often used in combinatorial optimization problems. More generally, this investigation is the first to explore the role of experimental noise in chemical computing. The modality of the HCMC system allows for inherent inexpensive scaling, increasing the number of variables and complexity of the possible problems by simply increasing the number of working electrodes via the use of microelectrode arrays. Additionally, our experiments demonstrate that the experimental noise within the measurement is sufficient to solve not only 4-variable number partitioning problems but also 7-variable problems, such as prime factorization and 3-SAT. Thus, this work demonstrates the use of key molecular subsystems as part of a functional HCMC. This demonstrative HCMC opens the way to more complex computational problems that take advantage of chemical behavior and development of more fully molecular implementations.

## Supporting Information Available

Experimental details, materials and methods for reaction solutions, noise experiments, additional HCMC concepts and coding details. Additional HCMC computations, including 2-SAT and 3-SAT. Simulation of electrochemical properties of HCMC (PDF)
Supplementary Movie showing the fluorescence response from Sites 1–7, with colorized overlay indicating the intensity ratio between the two imaging channels (AVI)

## Supplementary Material

oc3c00515_si_001.pdf

oc3c00515_si_002.avi
