# Supplementary material for: The Role of Experimental Noise in a Hybrid Classical-Molecular Computer to Solve Combinatorial Optimization Problems
Source: ACS Cent Sci. 2023 Jul 14;9(7):1453–65. doi: 10.1021/acscentsci.3c00515 (PMC10375572; doi:10.1021/acscentsci.3c00515)
Supplement: Supplementary file 1 — oc3c00515_si_001.pdf [file oc3c00515_si_001.pdf]

The Role of Experimental Noise in a Hybrid Classical-Molecular Computer to Solve Combinatorial Optimization Problems

Veronica K. Krasecki[a], Abhishek Sharma[b], Andrew C. Cavell[a], Christopher Forman[c], Si Yue Guo[d], Evan Thomas Jensen[a], Mackinsey A. Smith[a], Rachel Czerwinski[a], Pascal Friederich[d], Riley J. Hickman[d], Nathan Gianneschi[c], Alán Aspuru-Guzik[d], Leroy Cronin[b]*, Randall H. Goldsmith[a]*

[a] Department of Chemistry, University of Wisconsin-Madison, Madison, WI, 53706, United States

[b] Department of Chemistry, University of Glasgow, Glasgow, G12 8QQ, United Kingdom

[c] Department of Chemistry, Northwestern University, Evanston, IL, 60208, United States

[d] Department of Chemistry, University of Toronto, Toronto, Ontario, MS5 3H6, Canada

Corresponding Author

* Leroy Cronin, Email: Lee.Cronin@glasgow.ac.uk

* Randall H. Goldsmith, Email: rhg@chem.wisc.edu

1 Safety Statement

No unexpected or unusually high safety hazards were encountered. Free space lasers at high powers can pose risks for eye damage. The laser, along with the full optical setup, was contained in a custom-made enclosure to prevent hazards from scattered light and accidental reflections. For additional precautions, individuals could purchase laser safety glasses specified for specific wavelengths used.

2 Preparation of Chemical Reaction Solutions

A stock solution of 30% Pluronic F-127 (Sigma Aldrich) was made in Millipore ultrapure water. The solution was prepared at cold temperatures and stored in a 5°C fridge. Stock solutions of 100 mM p-Benzoquinone (Sigma Aldrich) and 100 mM Hydroquinone (Sigma Aldrich) were prepared with 27% Pluronic F-127 solutions. 2 mM stock solution of SNARF (5-(and-6)-Carboxy-SNARF-1, ThermoFisher Scientific, Invitrogen) was made in Millipore water. Finally, for the buffer, 1 M stock solutions of potassium phosphate monobasic and dibasic potassium phosphate were made in Millipore water. These solutions were combined to create a 1 M pH 7 phosphate buffer.

The stock solutions above were used to make the reaction solution. The reaction solution was made by layering p-Benzoquinone (23 mM), then 30% Pluronic solution, Hydroquinone (23 mM), and then adding SNARF (180 μM) and phosphate buffer (9 mM). The reaction solution was kept cold on ice before using. 100 μL of the reaction was placed on the electrode chip for use in HCMC. The electrode chip and reaction solution were heated for 30-60 seconds at 30°C before placing a

top coverslip on the sample and pressing the reaction solution to a thickness of 0.56 mm by using glass spacers.

3 Optical Experimental Set Up

An open home-built epifluorescence microscope was used for the HCMC. The optical set up consists of a 40 mW 488 nm laser (Coherent Sapphire). The beam size is expanded 30×. A 505 nm dichroic (Semrock) is used to reflect the excitation light onto the sample through a 1× air objective (Nikon, Plan UW, NA 0.04). The electrode chip sits on a metal stage within a 3D printed holder to ensure the sample position is reproducible for each implementation of the computer. The metal stage sits on a translation stage which controls the z-axis and allows the sample to be brought to focus. The fluorescence is collected via the same 1× objective, passing through the 505 nm dichroic, and is directed towards the detection setup. The emission is spectrally isolated from the laser light and background fluorescence using a 532 nm long pass filter (Semrock) and 540 nm long pass filter (Semrock). The emission is then spectrally split into two channels using a 610 nm dichroic, (Semrock) and then focused onto two separate cameras (Thorlabs, DCC1545M). Image acquisition was done through a custom written LabView code. Images were taken sequentially at each camera with a 1 second exposure time.

4 Electronic Components

4.1 Electrode Array Design

A key component of the HCMC is an array of addressable electrodes that can interface with the electrochemical gel to transfer information between digital and chemical domains. For ease of simplicity and cost-effective design, we used larger electrodes (diameter 1 mm) such that the electrode arrays can be fabricated using standard PCB manufacturing. The electrodes were placed in a hexagonal grid to have higher nearest-neighbor connectivity, see **Figure S1(A)**. The region around the electrodes shown in the white circle is an active area and no connection vias were placed within the active circle to avoid short-circuiting. All the electrodes were gold-plated by the PCB manufacturer, and FFC connector was used for electrical contact with the electrodes. The PCB-based electrode arrays were designed using Altium Designer Ltd. An additional board called as Pin Expansion Board was also designed for the ease of interfacing the electrode array with the standard electronic power supply or potentiostats, where each FFC connector is mapped to standard pin headers, see **Figure S1(B)**. All the experiments in this work were performed using PCB-based electrode arrays. All the design files for the electrode array and pin expansion board to connect to the electrochemical potentiostat including schematics, PCB design, and the exported Gerber files are available online at https://github.com/croningp/HybridComputation.

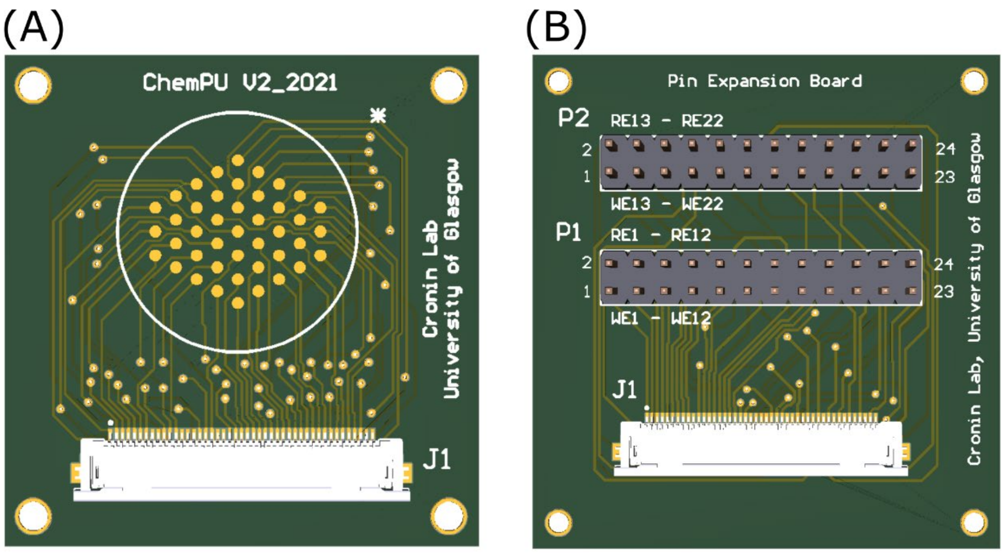

Figure S1. PCB-based electrode array and pin expansion board. (A) shows the design of PCB based electrode array with a hexagonal network of gold electrodes. The active area is within the white circle without any vias within that region. The electrodes are interfaced with a FFC connector. (B) shows a pin expansion board which connects FFC connector to standard pin headers for all electrodes.

As an extension to the current electrode array design, we also conceptualized a new electrode array design switching from the PCB-based electrode array to Si-wafer-based gold electrode array where the electrode size can be easily miniaturized to a few microns using standard lithography techniques. In addition to the working electrodes, it also consists of localized reference electrodes in the vicinity of each working electrode. Examples of the electrode array designs with local reference electrodes are shown in **Figure S2** with square and hexagonal grids. The working disk electrodes are surrounded by four connected mini-reference electrodes which can be coated with Ag/AgCl. The presence of local reference electrodes for each working electrode is useful when localized compartments, such as droplets, can be created over each electrode resembling an interconnected network of localized electrode-electrolyte pairs.

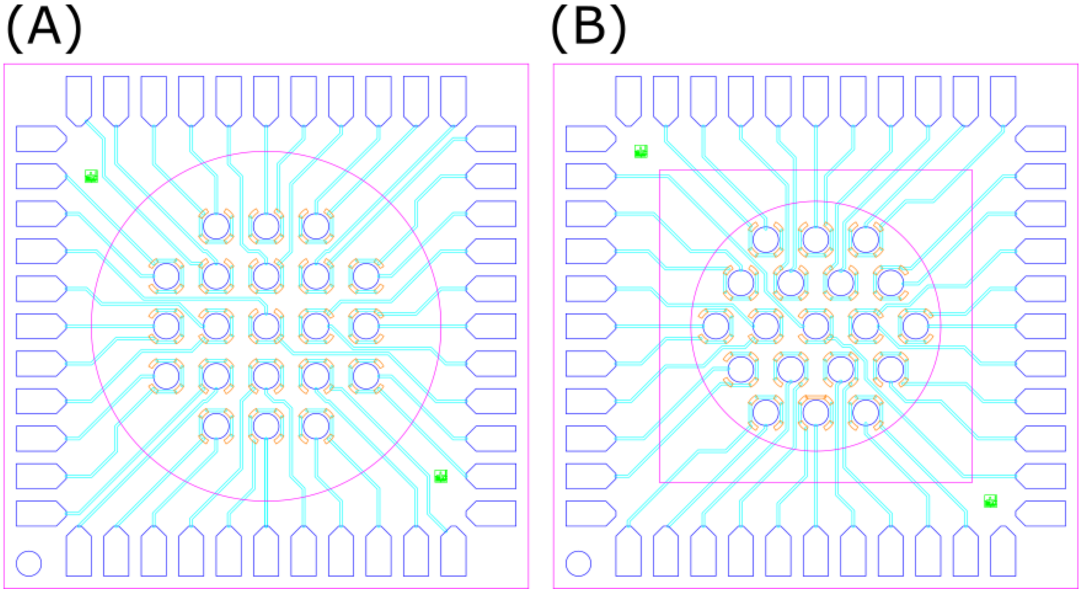

Figure S2. Designs of electroactive arrays on Si wafer. (A) and (B) shows designs of electroactive arrays in square and rectangular grids with local reference electrodes.

The proposed electroactive arrays can be directly glued on a PCB such that all the electrodes connected to the contact pads can be wire-bonded to the PCB contacts and all the electrodes are accessible using a FFC connection. The rendered design of the electrode array and PCB to create a complete device is shown in **Figure S3**. All the design files for 4-inch wafer scale electrode arrays (photomasks for gold connections and openings) and well as for PCB design to manufacture the complete assembly of the electroactive arrays are available online at https://github.com/croningp/HybridComputation.

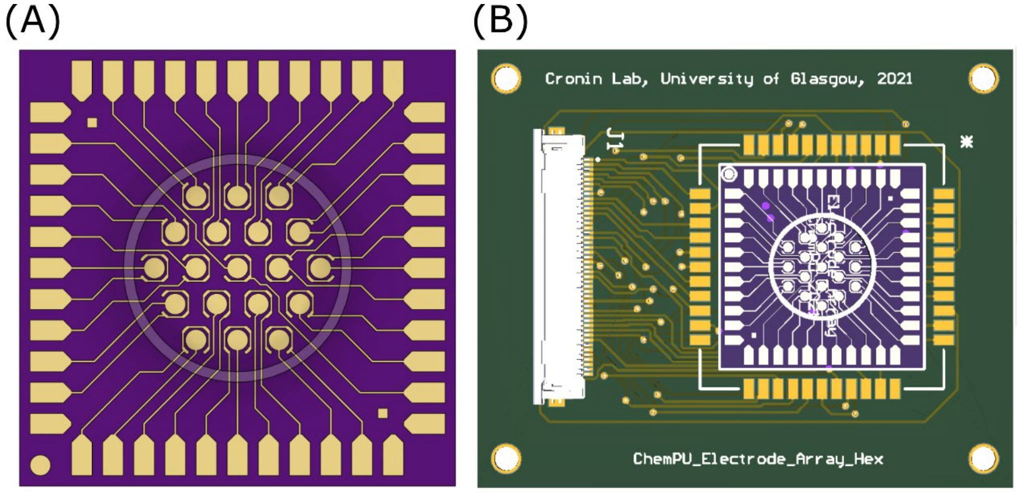

Figure S3. Wafer based electrode array with PCB mount. (A) shows a design of wafer-based electrode array (gold on silicon). Each working electrode (disk) is surrounded by four mini reference electrodes. The electrodes connected to the surrounding contact pads that can be wire-bonded to a PCB. (B) PCB design to mount a wafer-based silicon array with contact pads bonded to PCB using wire-bonding. The PCB can be connected to potentiostat controller using FFC cable.

4.2 Potentiostat

The electrode chip is connected to a multi-channel potentiostat (Rodeostat, IO Rodeo), which had some minor alterations to the publicly available firmware; specifically, the multiplexed electrodes are not set back to ground and allowed to float during times when they are not directly controlled by the multiplexer, this gave a better regulation of pH than otherwise. For this implementation of the HCMC, the potentiostat is connected so that there are 7 working electrodes along with 7 counter electrodes. In this implementation, the reference lead is connected to the counter electrodes, thereby resulting in a 2-electrode electrochemical cell rather than a 3-electrode electrochemical cell.

5 pH Fluorescence Calibration

1 M stock solutions of dibasic potassium phosphate and potassium phosphate monobasic were used to make multiple solutions of pH values varying from 5.5 to 7.5. The pH was checked and confirmed using a pH probe (Mettler Toledo). Reaction solutions were made with each pH buffer. 100 µL of reaction solution at a thickness of 0.56 mm was imaged for 100 seconds. The electrode chip was not connected to the potentiostat at this time, as no potential was applied. To calculate the intensity ratio corresponding to each pH, a region of interest was selected over each electrode. The intensity at each of these sites was measured and then the intensity at Camera 1 was divided by Camera 2 to obtain the intensity ratio. This was done at each electrode for each pH. Calibration curves of this information can be seen below in **Figure S4**. The pH range for the -1 to +1 states for each electrode are listed in **Table S1**.

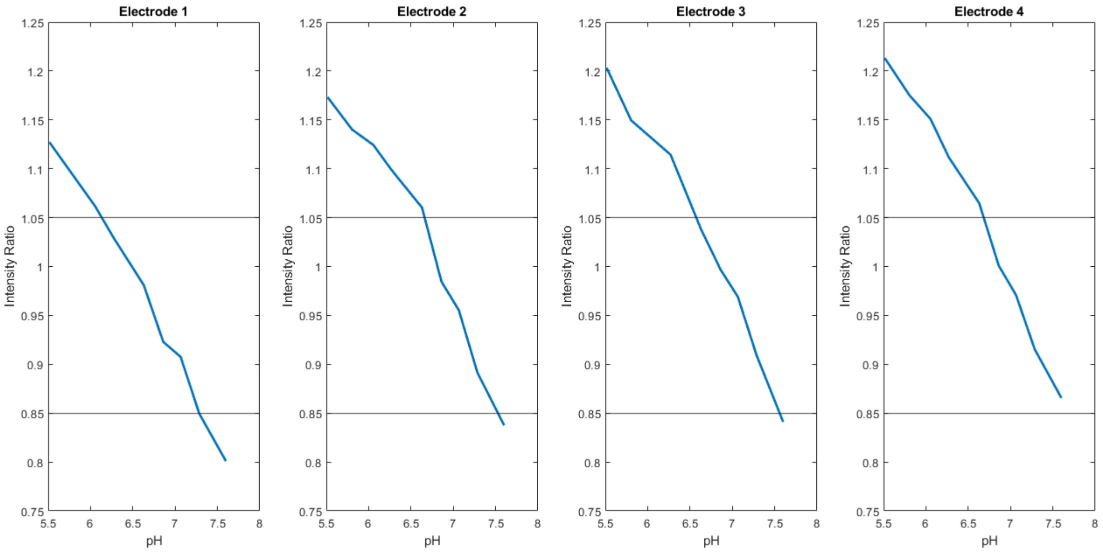

Figure S4. Calibration of pH to intensity ratio over the four electrodes used in the HCMC. Horizontal lines at IR values of 1.05 and 0.85 correspond to the intensity ratios for the +1 and -1 state values.

Table S1. pH values for each electrode

Electrode Number	pH for -1 State	pH for +1 State
1	6.13	7.28
2	6.66	7.53
3	6.57	7.56
4	6.68	7.7

6 HCMC Conceptual Context

In general, any physical system can compute if the accessible states of the system are isomorphic to an abstract representation of mathematics[1]. For example, a chemical computer requires the potential energy landscape[2,3] of the chemical system to be isomorphic to a mathematical problem[4]. In this way, the natural Brownian motion through the physical states allows exploration of mathematically associated logical states[4]. In practice, constructing a chemical system to have a specific potential energy landscape is extremely challenging, which makes programming a chemical computer, a seriously difficult problem in itself! However, by selectively applying electrochemical potentials to a site's electrode, a pseudo or umbrella landscape can be imposed on top of the native Potential energy surface, in which case the behavior of the droplet depends in a nonequilibrium fashion on both its native energy landscape and behavior of the electrochemical potential. In essence the electrochemical potential drives the chemical droplet to explore a specific sub-set of molecular configurations that have a similar pH.

Each possible configuration of the atoms in the chemical site will have a specific pH, and so the pH is a useful order parameter onto which the full potential energy landscape of each site is projected, and the electrochemical driving force can select specific trajectories. There is a large entropy associated with each specific computational state, i.e. each possible pH value has many configurations associated with it, and energy must be continuously applied and dissipated into the environment to keep the system exploring only states that yield that specific order parameter. Thus, the full range of configurations that the physical site can occupy is indexed by the pH.

In a fully molecular computer[5], as opposed to the HCMC, droplets would interact with each other entirely chemically, and the classical computer would be removed from the equation entirely. In that case each droplet would mutually affect their neighbor's behavior, in full analogy with a spin lattice. Programming/engineering such a fully molecular computer comes down to tuning the interaction strengths between each droplet individually, so the overall coupled behavior of the entire chemical droplet system in a full computer yields a single potential energy landscape across the entire set of molecules in all the droplets that is strongly isomorphic to the desired cost function. In the full computer it makes sense to refer to the energy landscape of the chemical system and the scalar cost function of the problem Hamiltonian, as being identical. However, in stark contrast, the cost function in the HCMC exists solely within the classical computer and is imparted to the chemical droplets though its electrode and the corresponding umbrella potential. The internal self-interaction of each droplet—i.e. its internal response to the electrode potential—is part of the

computation also, but in our case we have selected a chemical system that responds reversibly in a one to one mapping to the applied potential in a linear way. However, the possibility of supplying information in the form of auxiliary chemical titrations or light to such droplets is a very real possibility that would have an algorithmic effect on the computation thus encoded.[5,6]

7 HCMC Coding Details

7.1 Hamiltonian Generation

A Hamiltonian is used to encode the computational problem solved by the HCMC. For physical systems, the Hamiltonian describes its energy function; here, it represents the cost function of the optimization problem. The scalar cost function defined by the Hamiltonian over the state space is referred to as "energy" in this work. This scalar cost function (or energy) is what we are exploring in our computations. As explained in the main text, we rely on the mapping of combinatorial optimization problems to the Ising model for magnetic properties of two-state spins. Therefore, for each category of computational problem listed below, the Hamiltonian is expressed in a form analogous to the Ising Hamiltonian, namely with terms corresponding to a local field and to interactions between pairs of variables. (See Ref. 7 for a comprehensive list of Ising formulations of NP problems.) In the following paragraphs, we provide descriptions of the specific problem Hamiltonians used in this work.

7.1.1 Number Partition

The number partitioning problem asks: given a set of positive numbers n_i, i = 1, …, N, is there a way to partition the set into two disjoint subsets such that the sum of the numbers in both sets is equal? It is an NP complete problem with a direct mapping to the Ising Hamiltonian: for each number in the set, n_i, assign a spin (or binary) variable s_i, such that $s_i = +1$ if it is in one subset and -1 if it is in the other subset. The Hamiltonian is then given by $H = (\sum_{i=1}^{N} n_i s_i)^2$, the sum is squared to ensured nonnegative energy values. The solution is reached when H=0.

The assignment of state +1 or -1 to a subset is arbitrary and doubles the number of solutions. Therefore, without loss of generality, we can assign $s_1 = +1$ for the first number of the set. This reduces the quantity of binary variables for this problem by one, to N-1 for a set of N variables. In the HCMC demonstration presented in the main text, the four working electrodes, each corresponding to one variable, can solve a partitioning problem of five numbers.

7.1.2 2SAT and 3SAT

Another important problem is Boolean satisfiability (SAT)[8] which is NP complete for Boolean formulas with three or more literals per clause (k-SAT, k>=3). 2-SAT is the easiest case and can be solved in polynomial time. For the HCMC, we considered 2-SAT and 3-SAT cases and assigned each site in the HCMC to one of the variables within the clauses of the problem.

As in the number partitioning problem, the problem Hamiltonian is constructed by assigning each Boolean to an Ising spin s_i and evaluating the Boolean formula in an Ising Hamiltonian derived from its conjunctive normal form. The +1 and -1 states represent *true* and *false* values respectively. For example, if given the clause (1 and NOT 2), the site above Electrode 1 will be the variable "1" in the clause, and the site above Electrode 2 will be the variable "2" in the clause. The state value at each of these sites would tell us if the variables had to be true or false to make the clause satisfiable (said another way, correct). For this example, Site 1 would have to be true (+1) and Site 2 would have to be false (-1) to make the overall clause true (1 (true) and NOT 2 (false) is true). Details of the mapping of Boolean satisfiability to the Ising Hamiltonian, as well as its implementation on a molecular computer can be found in Ref. 5.

7.1.3 Prime Factorization

Prime factorization is an important problem in cryptography and cybersecurity which aims to identify all prime factors for a given number N. In this work, we considered a simplified version of this problem where the given number is a biprime, or a product of two primes and the method identifies its two odd factors, p and q, assumed to be prime.[9–11] Representing biprime factorization as an Ising Hamiltonian begins with expressing all numbers, the biprime and its yet-to-be-solved factors, as a binary numbers. One spin variable is needed to for each digit in the binary representation of the factors, p_i and q_i.

For $N = pq$, $N = \left(n_{n_N} \dots n_2 n_1\right)_2$, $p = \left(p_{n_p} \dots p_2 p_1\right)_2$, and $q = \left(q_{n_q} \dots q_2 q_1\right)_2$
$N = \sum_{i=1}^{n_N} 2^i n_i$, $p = \sum_{i=1}^{n_p} 2^i p_i$, and $q = \sum_{i=1}^{n_q} 2^i q_i$

The Hamiltonian is then constructed by expanding $H = (N - pq)^2$. (Since p and q are odd numbers, $p_1 = q_1 = 1$.)

For the HCMC with seven electrodes, we can solve problems $p = (p_5 p_4 p_3 p_2 1)_2$ and $q = (q_4 q_3 q_2 1)_2$, or up to a maximum of $N = 31 \times 15 = 465$.

Solutions to the prime factorization problems are expressed in binary, with each binary value corresponding to the state at each site. In contrast to number partitioning, where the state defined groupings, these solutions comprise of seven numbers which can either be +1 or -1, that are split to signify the two factors, with the first four representing one number and the last three representing the second number. To allow factoring of large numbers (upper limit of n = 465), we've shorted how the solutions are expressed. As all factors are odd and should end in a 1 in binary, the last number 1 is removed from the problem by fixing its value to +1. For example, when factoring 91: 7*13 results in 91, 7 is expressed in binary as [**111**] and 13 in binary is expressed as [1101]. Instead of the solution being [**111**1101], as the last number one's are removed, the solution is [**0011**110]. There is a second solution as well, for 13*7 which is [0110**011**]. As the HCMC values for the sites

range from +1 to -1, the solution expressed as states for the 7 electrodes would be [-1,-1,1,1,1,1,-1] and [-1,1,1,-1,-1,1,1], with 0 being represented as -1.

7.2 Image Processing

Joint analysis of a pair of images taken from the two spectroscopic channels determines the local pH of the gel above each electrode. For each electrode two pixels are selected manually indicating the sure foreground and the edge of the sure foreground, as well as indicating which electrodes are active on this run. The watershed algorithm, implemented in OpenCV, then segments the image into indexed regions of interest (ROIs) that pertain to the specific electrodes. The average intensity across the ROI for each electrode is computed in each of the two channels using the same ROI for both. The ratio of the intensities (IR) is linearly related to pH. Every image is scaled by the same global factor determined by the max and min values of intensity in the first image of the sequence to ensure the full range of observed intensities lies within the data scale.

7.3 PID

The PID loop is a standard feedback control system for tracking the electrochemical potentials on the electrode array. The IORodeo potentiostat is set up in multiplex mode, and each channel has its own trio of PID gains (k_p, k_i, k_d). Each time the main python control code takes a step in the gradient descent it calls the PID update procedure which cycles between the selected multiplexed channels, until the IR measured at each electrode reaches the desired set point. The voltage applied to each electrode is computed by maintaining a history of the error between the current IR and a setpoint IR for each electrode. The error, error sum and difference between the current and previous IR error are combined with the three gains to yield an updated voltage, as shown in Equation 1. The new voltage is clipped to the maximum and minimum allowed. Once all the IRs settle at their set point, the PID controller relinquishes control to the rest of the main loop. The PID gains are tuned by means of a standard test (simple jump in set point), to ensure that the PID does not overshoot too far or oscillate excessively before stabilizing.

$$V(t_i) = k_p \in (t_i) + k_i \sum_{j=0}^{i} \in (t_j) + k_d(\in (t_i) - \in (t_{i-1})) \tag{5.1}$$

Where $V(t_i)$ is the voltage at the time t_i of the ith cycle, $\in (t_i)$ is the measured error in IR vs a setpoint at the time of the ith cycle and (k_p, k_i, k_d) are tuned to have values that enabled rapid transition to a new value with critical damping.

7.4 Gradient

7.4.1 Gradient Descent Tests

The following was done to ensure the proper function of the gradient descent algorithm, i.e. correct prime factors were found purposefully (not converging coincidentally on correct solutions). The gradient descent algorithm was used to solve the factorization of n = 135 solely *in silico* at various step sizes and added noise parameter combinations. Step size values used were: 0.0001, 0.0005, 0.001, 0.005, 0.01, 0.05, 0.1, 0.15, 0.25, 0.5, and 1. Each of these step sizes were used in gradient descents with noise parameters of 0, 0.0001, 0.0005, 0.001, 0.005, 0.01, 0.05, 0.1, 0.15, 0.25, 0.5, and 1. Each pair of values was used 500 times. Each run was allowed to run for 500 steps or until the convergence criteria for the gradient descent were met. A graph of the results compiled from these 66,000 runs is shown below, in **Figure S5**. While almost all possible states (distributed along the x axis) are visited at least once in this data set, only a small subset were settled on by reaching the set convergence criteria. Of those that converged, some were incorrect solutions (shown in yellow) which correspond to local minima or plateaus. Correct solutions (shown in green) were also converged upon in a large subset of the runs. This work shows that the convergence on solutions is not random, as there are specific solutions that are preferred over others. While the distribution shown indicates that incorrect solutions (local minima/barren plateaus) were reached most of the time, it is clear that the correct solutions, again, are significant and not reached coincidentally. It is worth noting that the data is compiled from runs using a wide variety of parameters, and that the ratio of correct to incorrect final states could be improved here simply by using a single properly tuned set of noise and step size parameters in the actual computational runs.

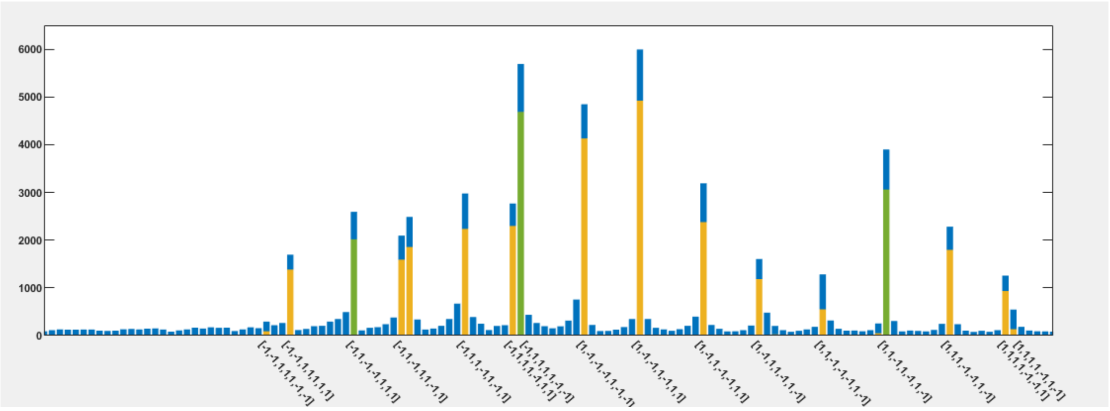

Figure S5. Histogram of test runs compiled from all conditions combined. Each state combination is visited at least once, but only local minima and correct solutions are converged upon (shown in yellow and green, respectively).

8 Generating Ising Hamiltonian Cost Function Landscapes

The landscapes shown in Figures 5 and 7 (in the main text) were generated to better visualize the paths taken by the gradient descent algorithm and identify minima. To this end, the problem Hamiltonian is used to calculate an energy value for each combination of state values and for a

range of intermediate state vectors. The resolution used in this work was 0.05, meaning each intermediate step is 0.05 away from the last step in state space. A Python script was used to generate all combinations of state values for 4 or 7 electrodes and calculate the energy for each one using the problem Hamiltonian. This dataset could then be used to plot surfaces with height corresponding to energy values for any set of 2 electrodes.

While the cost function for the Number Partitioning problem (Figure 5 in main text) is symmetric, it was observed that solution B was preferred when using the HCMC at Mode 1 when starting at Initial State 2. In this case, the descent finds the saddle point and oscillates in it for a long time before eventually falling to the minimum labeled B. The symmetry of the landscape should keep the descent algorithm stuck in the saddle; however, it falls to one side, and to the same side consistently. This appears consistent with a repeatable loss of numerical precision in our implementation, in which a slow magnification of the error eventually makes the system fall out of the saddle point.

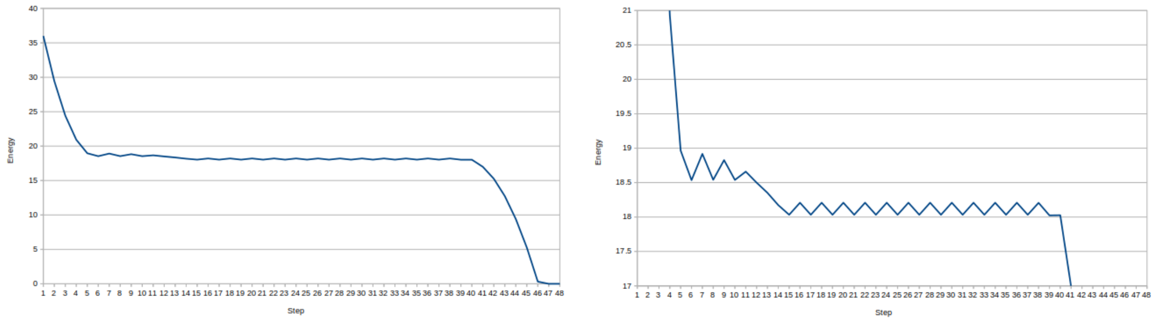

Figure S6. Value derived from the cost function during gradient descent using Mode 1 and Initial State 2. a) The values fall quickly to then oscillate around the center value of the saddle, but rather than infinitely repeating, they eventually fall to a lower energy corresponding to solution B. b) zoomed-in view of the intermediate oscillations.

9 2SAT 4 Electrode

Before using the hybrid computer to solve number partitioning problems, a simpler 2SAT problem was explored to vet the ability of the system. The 2SAT problem chosen used 4 sites (variables) and 9 clauses.

The input 2SAT clauses are as follows:
[‘1 or 3’, ‘2 or not4’, ‘2 or 3’, ‘1 or not4’, ‘1 or 2’ ‘not1 or 2’, ‘3 or not4’, ‘not3 or 4’, ‘1 or 4’].

Expressed in conjunctive normal form:

$$(1 \vee 3) \wedge (2 \vee \neg 4) \wedge (2 \vee 3) \wedge (1 \vee \neg 4) \wedge (1 \vee 2) \wedge (\neg 1 \vee 2) \wedge (3 \vee \neg 4) \wedge (\neg 3 \vee 4) \wedge (1 \vee 4)$$

The Ising Hamiltonian for this problem is:

$$H = 2.25 - 0.75s_1 + 0.25s_1s_3 - 1s_2 + 0.25s_2s_3 - 0.25s_2s_4 - 0.5s_3 - 0.5s_3s_4 + 0.25s_4$$

$$H_0 = 2.25$$
$$H_1 = [-0.75 \quad -1 \quad -0.5 \quad 0.25]$$
$$H_2 = \begin{bmatrix} 0 & 0 & 0.125 & 0 \\ 0 & 0 & 0.125 & -0.125 \\ 0.125 & 0.125 & 0 & -0.25 \\ 0 & -0.125 & -0.25 & 0 \end{bmatrix}$$

This problem has two solutions: [1,1,-1,-1] and [1,1,1,1]. In this instance, a +1 represents a true value and -1 represents a false value, therefore for the first solution when variables (sites) 1 and 2 are true and variables (sites) 3 and 4 are false the 2SAT problem is satisfiable, as all the clauses are true. For the second solution, when all the variables are true then the 2SAT problem is satisfiable as well. An example of a completed run by the HCMC solving this 2SAT problem, at Mode 3, is shown below in **Figure S7.**

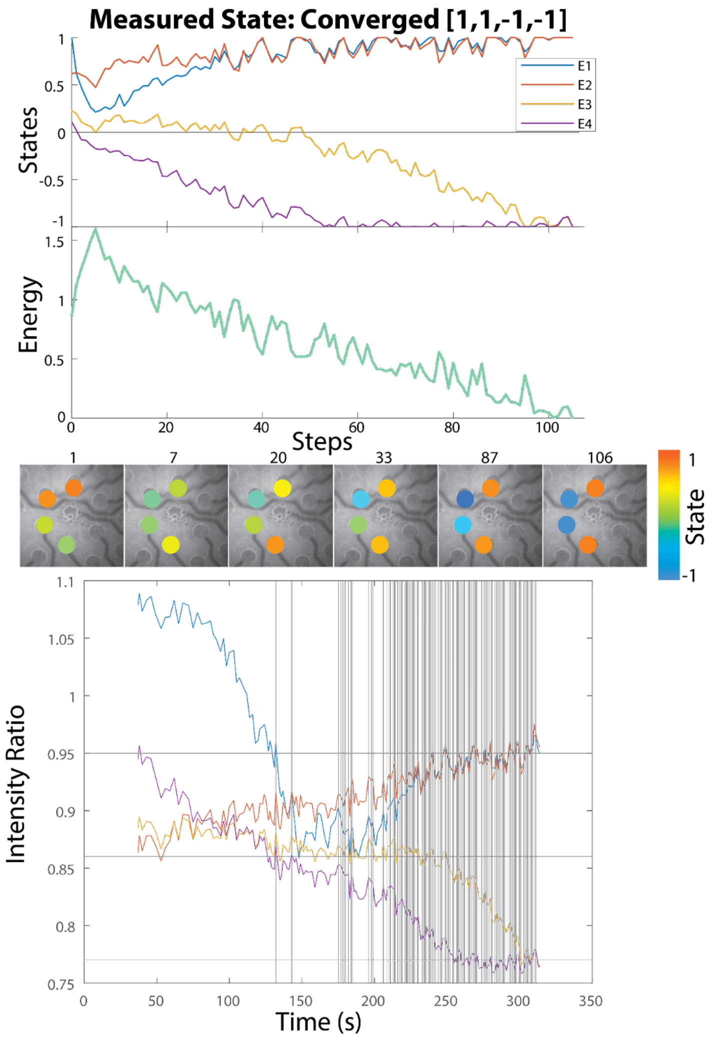

Figure S7. Progression of a computation by the hybrid classical-chemical computer solving a 2SAT problem. (Top) Evolution of states throughout the computation and energy of the Ising Hamiltonian at each step, where E1-E4 represent the four sites. (Center) Fluorescence images of the reaction gel on the electrode chip with artificially colored circles depicting the state value at various steps. (Bottom) The intensity ratios over time during the computation, vertical lines represent steps.

10 3SAT 7 Electrode

The HCMC was also able to solve a 3SAT problem using 7 electrodes (variables) with 28 clauses at Mode 3, using measured states with no *in silico* noise. The possible solutions that make this problem satisfiable are: [1,-1,-1,-1,-1,-1,-1] and [1,-1,1,1,1,-1,-1]. A completed and correctly converged run of the HCMC at Mode 3 solving the 3SAT problem is shown in **Figure S8**.

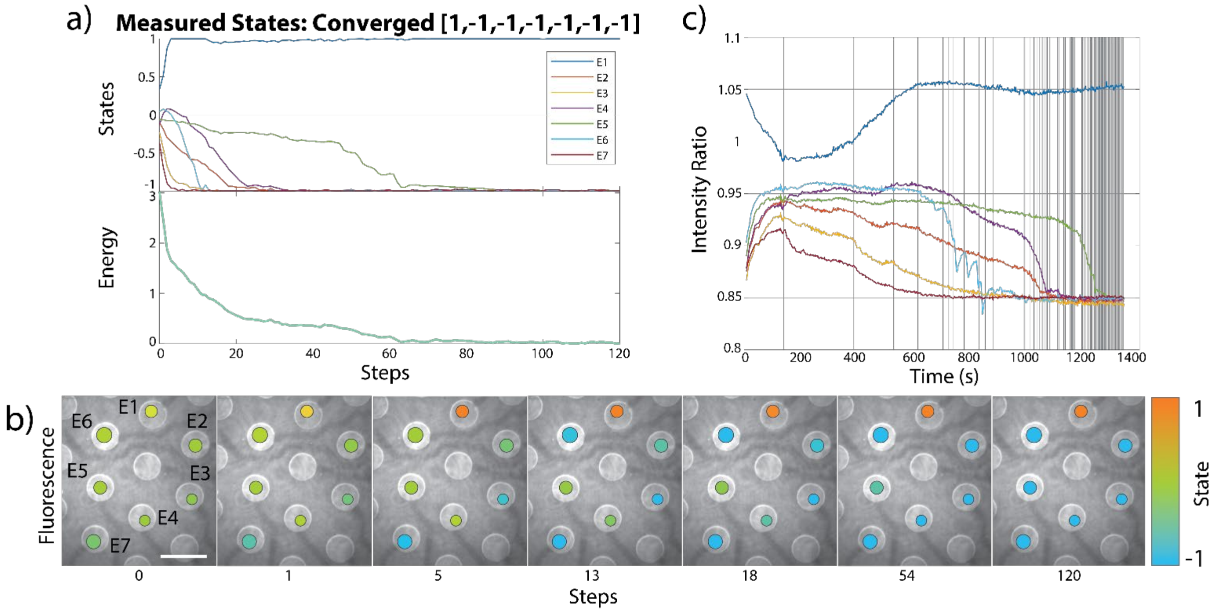

Figure S8. Progress of computation using the HCMC solving a 3SAT problem using 7 electrodes. (a) Evolution of states throughout the computation, where E1-E7 represent the 7 electrodes, and the Energy (Ising Hamiltonian scalar value). (b) Fluorescence image of the reaction gel on the electrode chip with artificially colored circles depicting the state value at various steps at each

11 Noise Measurement Experimental Details

11.1 Measurement of Noise in Applied Voltage

100 µL of reaction solution was placed onto the electrode chip, the solution is pressed to a thickness of 0.56 mm. To measure the potential, an external measure of the potential was performed using a National Instruments DAQ. Each electrode was measured individually by connected the DAQ to a single active working electrode along with the ground/counter electrodes. The potential was measured as each electrode was pulsed, applying a potential from 0V to -1V and then 0V to +1V. Additional measurements were performed where the potential was also pulsed at 0 to -1.4V and 0 to +2V. The potential measurement was acquired through LabView while simultaneously obtaining fluorescence images of the reaction gel. The measured potential was rebinned at 1 second intervals to be comparable to the HCMC settings. The signal after pulsing to +1 or -1V is taken and averaged. The standard deviation of the traces is calculated and then the mean and standard deviation are used to calculate the percent deviation. The noise in applied potential was found to be insignificant relative to the noise added to the experiment from the optical measurement, with the values reported in **Table S2**.

Table S2. Calculated Percent Deviation for Various Applied Potentials for the 4 Working Electrodes

Goal Potential (V)	Percent Deviation (%)
-1	0.03
+1	0.03
-1.4	0.02
+2.0	0.02

11.2 Measurement of the Noise in the Current

After confirming that the applied potential is stable (as per the previous section), we then proceeded to perform controlled-potential chronoamperometry experiments. 100 μL of reaction solution was placed onto the electrode chip, the solution is pressed to a thickness of 0.56 mm. The potential was held first at 0V to establish a baseline, then stepped to +/- 1V, -1.4V, or +2.0V and held for 5 minutes while measuring the current. This was repeated for each electrode in turn to account for any variation among them. The current was measured using the same multi-channel potentiostat used for the HCMC. Meanwhile, the imaging setup was used to record intensity ratios at each electrode during these runs to monitor for any apparent changes in pH over the course of these experiments. Any noise observed in these current measurements will be influenced by noise in the potential applied, fluctuations in current caused by interactions at the electrode surface, and noise in the measurement of the current itself.

11.3 Conversion of Current Noise to Fluctuations in Intensity Ratio

To compare the measured noise levels in the above section to the values from the optical noise measurements, the measured current was used to calculate a theoretical pH change over time. The fluctuations in this value could then be used to determine a theoretical IR noise value that would have resulted from any current fluctuations. This was done by assuming 100% Faradaic efficiency, such that any change in measured current corresponded to a change in quinone concentrations at the electrode surface, which would directly relate to gain or loss of protons. It is worth noting that this ignores any background current and any fluctuations that could arise from other sources. Though unrealistic, operating in this limit gives us a value for the maximum possible contribution from current noise to the overall measured noise in our computational runs.

To do this conversion, the current was converted to a net electron flow, which was converted directly into change or loss of proton concentration at the electrode surface (depletion of quinone at the surface was disregarded to get an upper limit on concentration changes). After accounting for buffering by our solution, this was converted to pH change over time (see **Figure S9**). Using our pH calibration, this could be directly converted to an intensity ratio (IR) as seen in

our experiments. The noise in this IR trace is then directly comparable to the noise in the IR measured experimentally. As shown, the maximum possible contribution to experimental noise from current fluctuations is still insignificant relative to the imaging noise.

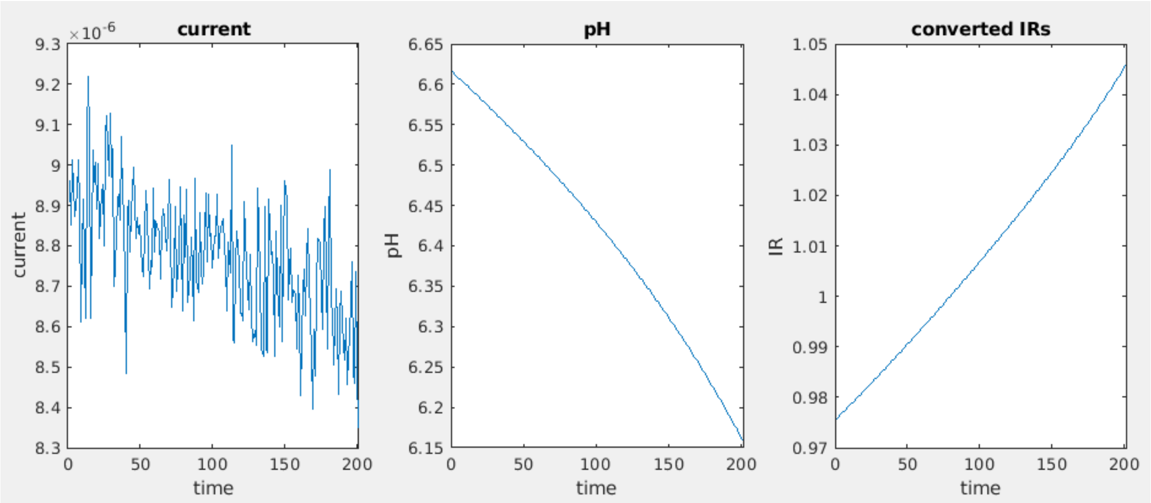

Figure S9. Current trace from noise measurement, corresponding pH values over time, and intensity ratios converted from these pH values.

11.4 Measurement of Noise in Laser

A digital optical power meter (Thorlabs) was used to measure the noise within the excitation laser source. With all other additional lights turned off, the sensor was placed in the beam path of the excitation laser and data was collected in 1 second intervals for 300 seconds. The measured signal was averaged together, and standard deviation and percent deviation were calculated. The average power was 41.79 mW (0.02), with a percent deviation of 0.06%.

11.5 Details of the Noise from Optical Measurement

After confirming a stable laser excitation, we then measured the noise resulting from our optical measurement. These experiments were performed by imaging the reaction solution on the electrode chip without any applied potential. The sample was excited by the 40 mW 488 nm laser. Image acquisition was performed using the same settings as the HCMC experimental details. 300 images were collected at 1 second exposure, this was repeated 5 times. The fluorescence data was processed using MATLAB.

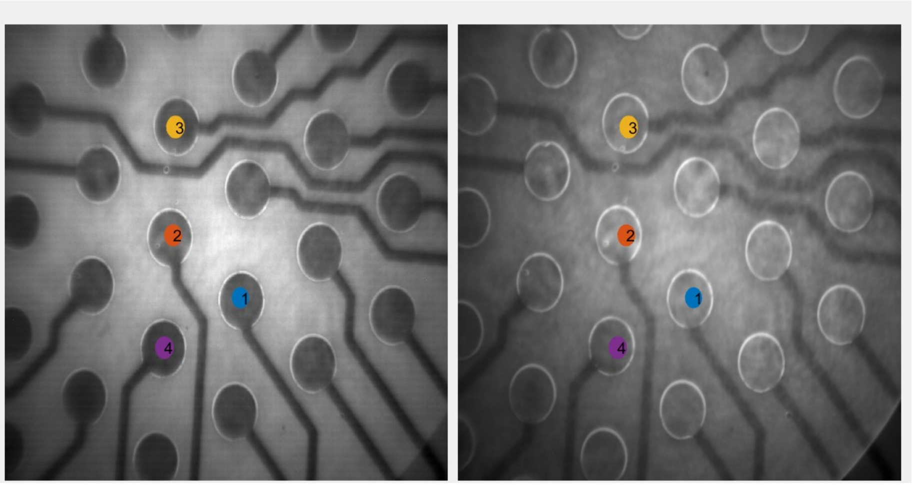

Figure S10. Fluorescence images of the reaction solution on an electrode chip. (Left) Channel 1 and (right) Channel 2, regions of interest (ROIs) in both channels are depicted by the colored circles over the electrodes, with the numbers labeling sites 1-4.

For each replicate the following was done, ROIs were selected at each site (over each active electrode) in both fluorescence channels, see **Figure S10**, and the intensity at each frame within the ROI was measured resulting in fluorescence intensity traces for each camera at each site. The fluorescence IR was calculated with Fluorescence Intensity Channel 1 divided by Fluorescence Intensity Channel 2 for each frame, which generated an IR trace. See **Figure S11** for example of signal traces. The average, standard deviation, and percent deviation were determined for each signal (fluorescence traces and IR traces). The average intensity ratio over these experiments was 1.04, with a standard deviation of 0.002 and a percent standard deviation of 0.2%.

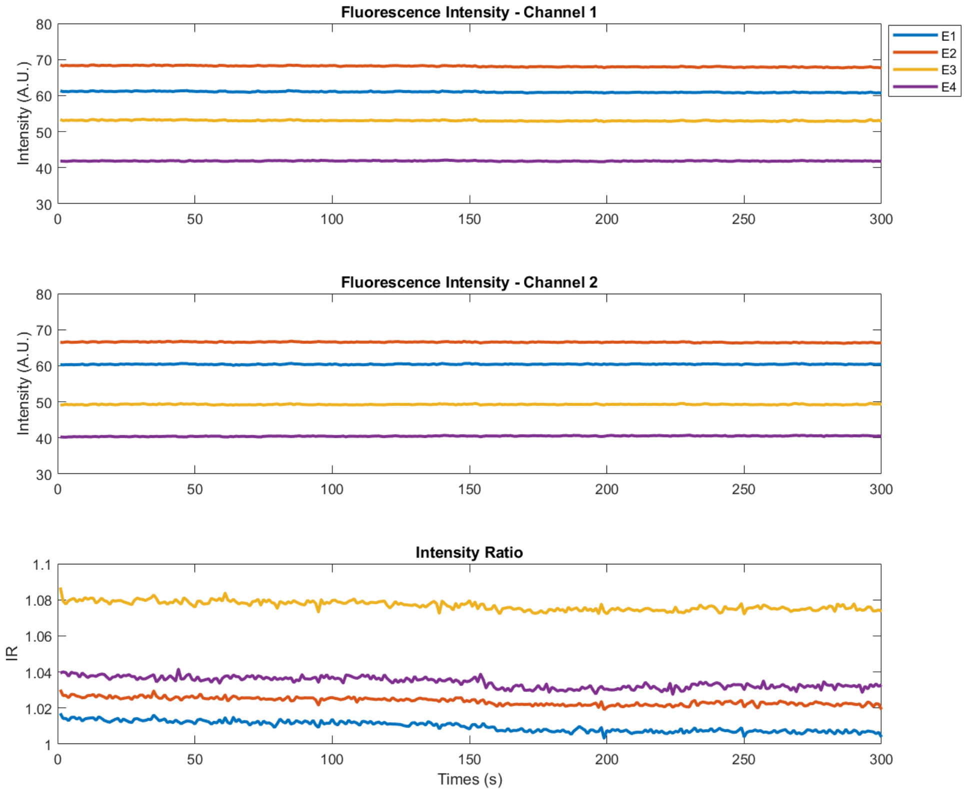

Figure S11. Plot of fluorescence intensity trace over time (s) for Channel 1 (top) and Channel 2 (middle). Plot of Intensity Ratio over time (s) (bottom). Each color trace represents a site over a different electrode.

11.6 Noise from Complete Experimental Measurement

The reaction solution was prepared as previously described and placed onto an electrode chip. The imaging acquisition is at the same settings previously stated. The reaction gel was imaged beginning at pH 7 and then potentials were applied (using the multiplexed potentiostat) bring each of the sites to a -1 state value, which corresponds to an IR of 0.85 and a more basic pH. The set IR was then maintained for 700 steps. This allows us to quantify the fluctuations from the full experimental set up under active PID conditions. For analysis, a section of the intensity trace after reaching the target IR was isolated (around 300 steps, see **Figure S12**) and used to calculate an average signal, standard deviation, and percent standard deviation, see **Table S3**. These experiments manipulated all 4 electrodes simultaneously. Additionally, the threshold for the PID was set to 0.01, which is the same value as the HCMC running at Mode 3.

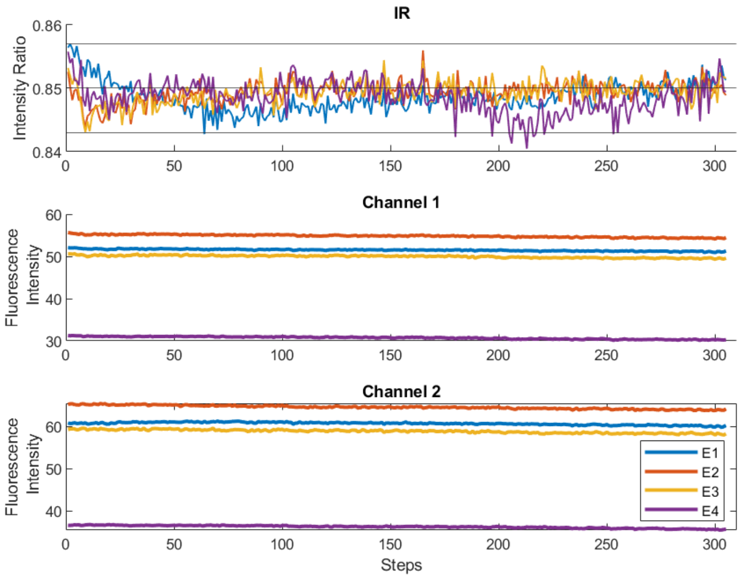

Figure S12. Plots of the IR (top) and fluorescence signals from Channels 1 (center) and 2 (bottom) while maintaining a set -1 state value using PID

Table S3. Compiled results from the complete experimental measurement.

**Run**	**IR**		
	**Average**	**Standard Deviation**	**Percent Deviation (%)**
**1**	0.850	0.002	0.24
**2**	0.850	0.002	0.26
**3**	0.850	0.003	0.32
**4**	0.850	0.002	0.18
**5**	0.849	0.002	0.24

12 Calculating Minimum *in silico* Noise

The following experiments were performed to determine the minimum noise necessary to not get trapped in a trajectory that converges on the local minima. The HCMC is solving the number partitioning Hamiltonian at Mode 2, completely *in silico*. The noise value (otherwise known as the standard deviation of normal gaussian) was varied by orders of magnitude from 0 (Mode 1 *in silico* noise value) to 1 (Mode 2 *in silico* noise value). Then additional runs were performed at *in silico* noise values of 0.005 and 0.02 (matching the experimental noise values). The distribution of solutions the computer converged on are shown in **Figure S13**. Each run began at Initial State III and there were 100 repeats at each set of conditions. The HCMC starts to converge on correct solutions (A and B) at a noise setting of 0.005, but still majority convergence on the local minimum (3% correct, 97% LM). When getting to 0.02 noise, the frequency of convergence at the LM reduces to 46%, with correct solutions being at 54% (split 30% A, 24% B). Finally, at Mode 2, there is more Solution A convergence than LM at all. When the *in silico* noise value was set too high, the stochasticity would cause extreme hopping between states and convergence on various

states, some that may be other local minima present within the energy landscape. Therefore, the *in silico* noise variable selected for Mode 2 was ideal to efficiently solve the number partitioning problem for these initial states.

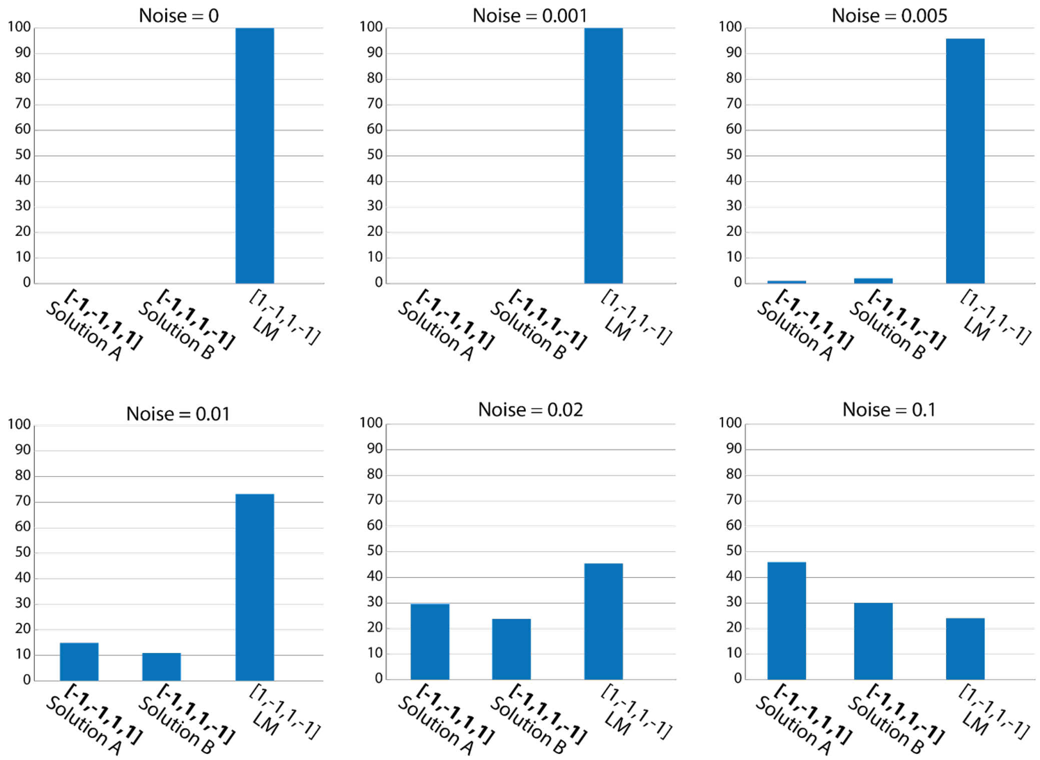

Figure S13. Bar graphs showing the propensity that a set of state values were converged on when running the HCMC at Mode 2) with varying *in silico* noise values (specified above each graph).

13 Simulations of Electrochemical Properties of HCMC

Details of the accompanying numerical simulation engine developed to describe the features of computation using the HCMC process are found below.

13.1 Hybrid Electronic-Chemical Computational Architecture

The generalized hybrid electronic-chemical computational architecture utilizes information processing distributed between digital and chemical substrates. In this architecture, the mathematical problem is instantiated into the digital computer and the hybrid algorithm utilizes both classical and chemical computation within a feedback loop, see **Figure S14**.

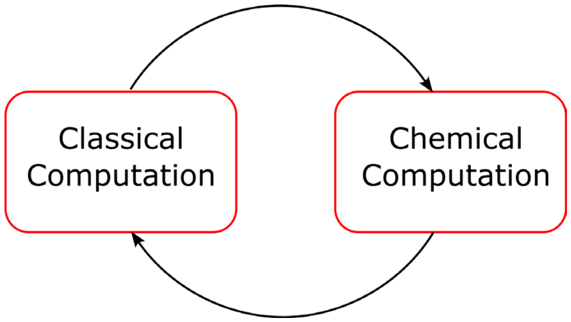

Figure S14. Representation of hybrid electronic-chemical logic.

The basic implementation of hybrid electronic-chemical computational architecture comprises a feedback loop between two information processing layers, 1. Digital electronic layer and 2. Analog Chemical Layer[12]. In the computational logic, at each step the information loops between the electronic and chemical layers as described in **Figure S15**. The overall logic can be represented by three states within digital and chemical domains and four state machines. The three computational states at time step t are defined as,

$\boldsymbol{CS^t_{a,ij}}$: Analog Interacting Chemical State in the chemical domain over the electrode e_{ij}
$\boldsymbol{CS^t_{d,ij}}$: Digital representation of Analog Chemical State after readout over the electrode e_{ij}
$\boldsymbol{VS^t_{ij}}$: Digital electronic state over the electrode e_{ij} representing the electrode potential.

The four state machines representing closed-loop information processing are given by,

F **Chemical Readout State Machine**: Analog readout of chemical state $\boldsymbol{CS^t_{a,ij}}$ based on ratiometric imaging of pH-sensitive fluorescent dye to its digital representation $\boldsymbol{CS^t_{d,ij}}$.

$$\boldsymbol{CS^t_{d,ij} = F(CS^t_{a,ij})}$$

D **Digital Finite State Machine**: A finite state machine logic which updates the digital electronic state $\boldsymbol{VS^t_{ij}}$ based on the digital representation of the chemical state $\boldsymbol{CS^t_{d,ij}}$.

$$\boldsymbol{VS^t_{ij} = D(CS^t_{d,ij})}$$

P **Digital-Chemical Interface State Machine**: Applies the digital electronic state $\boldsymbol{VS^t_{ij}}$ into the physical medium via the electrochemical interface (electrochemical kinetics)

$$\boldsymbol{\widetilde{CS}^t_{ij} = P(VS^t_{ij})}$$

C **Chemical State Machine**: Chemical state machine describing the interactions between electrochemical kinetics and buffer kinetics over the continuum chemical medium.

$$\boldsymbol{CS^{t+1}_{a,ij} = C(CS^t_{a,ij}, \widetilde{CS}^t_{ij})}$$

where, $\boldsymbol{\widetilde{CS}^t_{ij}}$ is the virtual chemical state which describes the effect of electrochemical kinetics that combines with the previous chemical state $\boldsymbol{CS^t_{a,ij}}$ to create a new chemical state. The complete

hybrid computational loop updates the analogue chemical state at step t, $\boldsymbol{CS_{a,ij}^{t}}$ to $t+1$, $\boldsymbol{CS_{a,ij}^{t+1}}$ as $\boldsymbol{CS_{a,ij}^{t+1}} = \boldsymbol{K}(\boldsymbol{CS_{a,ij}^{t}})$ where $\boldsymbol{K}$ represents the hybrid electronic-chemical state machine which is composed of digital processing together with chemical interactions as an outcome of electrochemical kinetics coupled with bulk chemistry such as buffer kinetics. The hybrid state machine can be described as,

$$\boldsymbol{CS_{a,ij}^{t+1}} = \boldsymbol{K}(\boldsymbol{CS_{a,ij}^{t}}) = \boldsymbol{C}\left(\boldsymbol{P}\left(\boldsymbol{D}\left(\boldsymbol{F}(\boldsymbol{CS_{a,ij}^{t}})\right)\right)\right)$$

or the hybrid state machine on general chemical state $\boldsymbol{CS}$ is given by $\boldsymbol{K}(\boldsymbol{CS}) \equiv \boldsymbol{C}\left(\boldsymbol{P}\left(\boldsymbol{D}\left(\boldsymbol{F}(\boldsymbol{CS})\right)\right)\right)$.

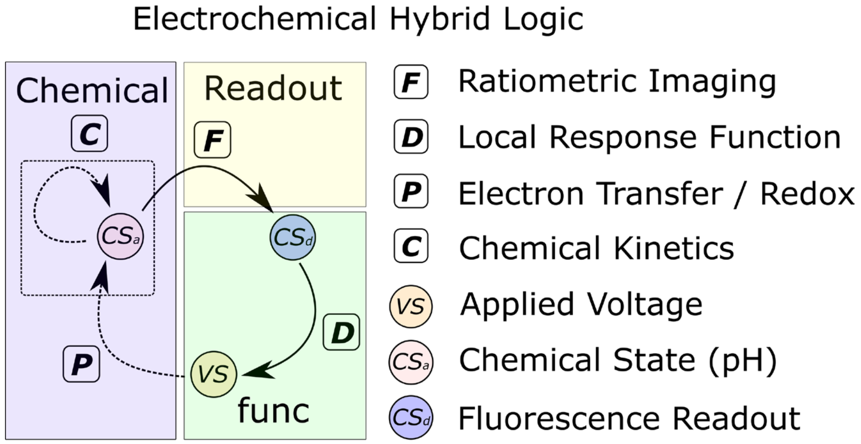

Figure S15. Representation of hybrid electronic-chemical logic. The figure shows the closed-loop logic of the hybrid electronic-chemical state machine $\boldsymbol{K}$. $\boldsymbol{CS_{a,ij}^{t}}$, $\boldsymbol{CS_{d,ij}^{t}}$, and $\boldsymbol{VS_{ij}^{t}}$ represents the three emerging states and $\boldsymbol{C}$, $\boldsymbol{P}$, $\boldsymbol{F}$, and $\boldsymbol{D}$ represent the four state machines.

The information transfer in a single loop between the digital and chemical domains is shown in **Figure S16**. In the first step, the readout of the Analog chemical state is based on fluorescence imaging of the dye molecules in the vicinity of the electrode. The ratiometric imaging of the Analog chemical state is then converted into a digital equivalent chemical state using a linear function, due to a well-defined linear relationship between intensity ratios and pH, see **Figure S16A**. As the next step, a pre-programmed digital Finite State Machine ($\boldsymbol{D}$) reads the digital representation of chemical state ($\boldsymbol{CS_{d,ij}^{t}}$) and updates the voltage level on the electrode (e_{ij}). This Finite State Logic is the core of digital information processing and uses a well-defined deterministic computational logic, see **Figure S16B**. This can also be used for basic computational operations such as complex logic gates, direct implementation of optimization problems in a physical substrate etc. On the application of voltage on the electrode, the new emerging chemical state is a combined outcome of the previous chemical state $\boldsymbol{CS_{a,ij}^{t}}$ together with the electrochemical kinetics on the application of voltage on the electrode (e_{ij}), see **Figure S16C**. To define the interaction of electrochemical kinetics with the previous chemical state, for simplicity, the

combined effect is quantified by two state machines $\boldsymbol{P}$ and $\boldsymbol{C}$, where the state machine $\boldsymbol{P}$ represents the effect of applied voltage at the interface leading to a virtual chemical state $\widetilde{\boldsymbol{CS}}_{ij}^{t}$ which then combines with the previous chemical state following state machine $\boldsymbol{C}$ to create a new chemical state $\boldsymbol{CS}_{a,ij}^{t+1}$. In the next subsection, we will describe the mathematical description of the physical system which is equivalent to the state logic defined by the four state machines.

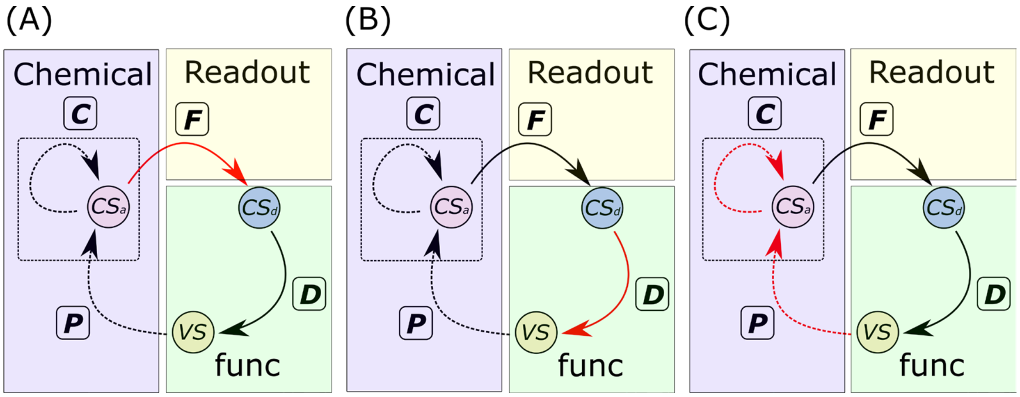

Figure S16. A single information loop with chemical and digital domains. The figure shows the closed-loop logic where information is looping between the four states machines, and the information transfer at each step is shown in red.

13.2 Electrode Network Model Using Secondary Current Distribution

The key idea is to develop a simple electrochemical network model to describe features similar to the experimental design such as pH control over the electrode array. In this section, we describe an electrochemical network model and the governing equations to simulate the dynamics of an interconnected network of localized electrode-electrolyte pairs. Due to the complexity of the experimental setup such as coupled electrochemical dynamics with bulk kinetics combined with multielectrode control logic, here we use a simplified model to describe a complete logic. Various parameters assumed in the simulation might not represent physical parameters from the experiments but given a clear description of the experimental design. By applying the time-dependent voltages on the electrode array, the simulation engine can predict time-dependent electrode currents, interfacial currents, and local pHs, which then can be utilized to create hybrid computation logic.

13.2.1 Governing Equations and Boundary Conditions

The experimental setup comprises a two-dimensional network of electrodes with a layer of gel electrolyte with redox-active species, together with buffer (such as phosphate) and the supporting electrolyte. We assume that the electrochemical and bulk kinetics that influence the computational logic occur locally in the vicinity of the electrode. With this assumption, we can define localized electrode-electrolyte pair as a circuit element comprised current source from the electrochemical

kinetics together with a set of resistors based on the conductivity of the solution and the geometric parameters. A single electrode-electrolyte pair is shown in **Figure S17A**. In this model, over a two-dimensional grid, on each electrode e_{ij} where i & j are the indices for nomenclature, electrochemical kinetics as a result of the applied potential leads to the current flow. The current flow is defined using a Secondary Current distribution model where the net current flowing through the electrode is given by the sum of double layer current and the Faradaic current. The electrolyte in the vicinity of the electrode is approximated by using a combination of bulk resistors: R_{B0} in the vertical direction and R_B in specific directions along the horizontal plane. The number of horizontal resistors depends on the connectivity between the electrode-electrolyte pairs. The electrode potential, electrolyte potential at the electrode surface, and electrolyte potential at the interconnecting horizontal surface for the electrode e_{ij} are designated as $\phi_{s,(i,j)}$, $\phi_{el,(i,j)}$, and $\phi_{p,(i,j)}$ respectively. The position of at which $\phi_{p,(i,j)}$ is defined depends on the geometry and in this work, we assume it at the top of the gel layer h_{el}. This is purely an assumption, and different possible heights (such as $h_{el}/2$) can be chosen based on geometric factors. This is not a crucial parameter as the resistance also depends on the bulk conductivity which can be tuned by changing the concentration of the supporting electrolyte. In the case of four neighboring electrode-electrolyte pairs, the edge of the electrode-electrolyte pair is defined at the radius of the electrode. The four potentials at the edge of the electrode-electrolyte pairs are given by $\phi_{pN(i,j)}$, $\phi_{pE(i,j)}$, $\phi_{pS(i,j)}$, $\phi_{pW(i,j)}$. The variables names N, E, S, W represents north, east, south, and west direction for the nomenclature in case of four nearest neighbors. The vertical electrode current and the four directional currents are denoted as $i_{el(i,j)}$, $i_{pN(i,j)}$, $i_{pE(i,j)}$, $i_{pS(i,j)}$, $i_{pW(i,j)}$ respectively. The resistance at the interconnection of the two electrode-electrolyte pair elements is defined as R_{int} and the currents flowing at the interface are equated to the four directional currents taking the directionality into account. The complete representation of the electrode array network comprised of local electrode-electrolyte pairs is shown in **Figure S17B**. A simplified example of the two coplanar electrodes with current potentials is shown in **Figure S17C**.

To define the voltage-dependent current flowing on each electrode in an array, we used Secondary Current Distribution Model where the Faradaic current due to electrochemical reaction is given by Butler Volmer kinetics,

$$i_{loc,e} = i_{0,e}\left(e^{\alpha_a \frac{F\,n\,\eta_e}{RT}} - e^{-\alpha_c \frac{F\,n\,\eta_e}{RT}}\right) \tag{13.1}$$

where, $i_{loc,e}$ is the local current density at the electrode e due to electrochemical reactions on the application of external voltage, $i_{0,e}$ is the exchange current density, n is the number of electrons transferred, η_e is the overpotential defined as $\eta_e = \phi_s - \phi_{el} - E^0$, α_a and α_c are the anodic and cathodic charge transfer coefficients, ϕ_s, ϕ_{el} and E^0 are electrode, electrolyte and equilibrium potentials, F is the Faraday's constant, R is the gas constant, and T is the temperature.

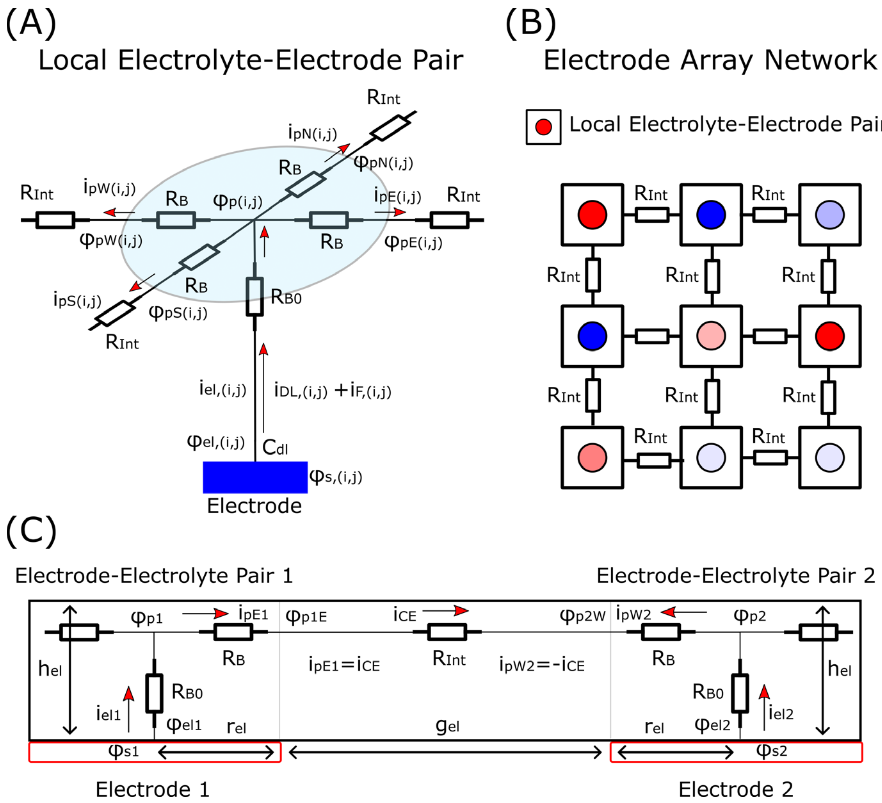

Figure S17. Representation of localized electrolyte-electrode pair network. (A) The figure shows an equivalent circuit model of localized electrolyte-electrode pair assuming four neighboring pairs. (B) An electrode array network is represented by interconnected local electrolyte-electrode pairs at different applied potentials. (C) A simplified example of electrolyte-electrode pairs from two coplanar electrodes.

The exchange current density is given by $Fk_0C_{el}^{\beta}C_{el}^{1-\beta}$ where C_{el} is the concentration of electroactive chemical species (oxidized and reduced species) considered as equal, β is the symmetry factor set as 0.5 and k_0 is the reaction rate. The total current (i_{el}) flowing through the electrode is equal to the sum of the double-layer current (i_{DL}) and the current flowing due to Faradaic reactions (i_F),

$$i_{el} = i_{DL} + i_F = i_{DL} + \sum i_{loc,e} \tag{13.2}$$

Substituting the double-layer current as $i_{DL} = C_{dl}\frac{d\eta_e}{dt} = C_{dl}\frac{d(\phi_s - \phi_{el} - E^0)}{dt}$ where C_{dl} is the double layer capacitance per unit area and Faradaic current $i_{loc,e}$, the current flowing through the circular electrode with a radius r_{el} is given by

$$i_{el} = \pi r_{el}^2 \left(C_{dl} \frac{d(\phi_s - \phi_{el})}{dt} + i_{loc,e} \right) \tag{13.3}$$

In the presence of multiple interconnected electrolyte-electrode pairs, the electrode currents on various electrodes can be combined with each other using Kirchhoff's current and voltage laws. As shown in **Figure S17A**, the conservation of the electrode current from the electrode e_{ij} splitting into four directional currents is given by

$$i_{el,(i,j)} = i_{pN,(i,j)} + i_{pE,(i,j)} + i_{pW,(i,j)} + i_{pS,(i,j)} \tag{13.4}$$

where, $i_{el,(i,j)}$ is the electrode current and $i_{pN(i,j)}, i_{pE(i,j)}, i_{pW(i,j)}, i_{pS(i,j)}$ are the four electrolytic currents in north, east, west, and south directions. Additionally, the voltage equations are given by,

$$\begin{aligned} \phi_{el,(i,j)} - \phi_{p,(i,j)} &= R_{B0} i_{el,(i,j)} \\ \phi_{p,(i,j)} - \phi_{pN,(i,j)} &= R_B i_{pN,(i,j)} \\ \phi_{p,(i,j)} - \phi_{pE,(i,j)} &= R_B i_{pE,(i,j)} \\ \phi_{p,(i,j)} - \phi_{pS,(i,j)} &= R_B i_{pS,(i,j)} \\ \phi_{p,(i,j)} - \phi_{pW,(i,j)} &= R_B i_{pW,(i,j)} \end{aligned} \tag{13.5}$$

where, $\phi_{el,(i,j)}$ is the electrolytic potential right above the electrode, $\phi_{P,(i,j)}$ is the electrolytic potential at the height above the electrode where currents become horizontal (currently assumed as the top of the gel), $\phi_{pN,(i,j)}$, $\phi_{pE,(i,j)}$, $\phi_{pS,(i,j)}$, $\phi_{pW,(i,j)}$ are the electrolytic potentials at the electrode edge of the electrode-electrolyte pair in north, east, south, and west directions. The bulk electrolyte conductivity for a symmetric $z{:}z$ supporting electrolyte is given by $\sigma_B = \frac{2z^2e^2C_SN_AD}{k_BT}$, where z is the ion valence, e is the electronic charge, C_s is the concentration of the supporting electrolyte, N_A is Avogadro's number, D is the diffusion constant, k_B is Boltzmann's constant, and T is the working temperature. We assume that the bulk conductivity of the solution is constant and is only governed by the supporting electrolyte. Using the bulk electrolyte conductivity, we can calculate the resistances: R_{B0}, R_B the vertical and horizontal resistances and R_{int} which is the interface resistance between two electrode-electrolyte pairs. The resistance R_{B0} is assumed as the resistance connecting the electrode to the height of the gel. So, the length of the resistance is h_{el} and cross section area is defined by the electrode area (πr_{el}^2). This is an approximation to quantify the vertical resistance from the electrode to the centre of the electrolyte assuming the current flows up to the complete height of the gel. As discussed previously, based on the geometry of the system such as the height, radius and electrode the effective length of the resistance defined by the height might be different. Similarly, R_B represents the horizontal resistance till the edge of the electrode. So, the length of the resistance is the radius of the electrode and the cross-section is assumed to be

rectangular with the area given by $2r_{el}h_{el}$. The width of the cross-section was assumed to be the diameter of electrode $2r_{el}$ and height as the height of the gel layer h_{el}.
Similarly, the horizontal interface resistance R_{int} between two electrode-electrolyte pairs is defined by effective length as gap length (g_{el}) and the cross-section defined by rectangular cross-section area given by $2r_{el}h_{el}$. Hence, all three resistances are given by,

$$R_{B0} = \frac{1}{\sigma_B}\frac{h_{el}}{\pi\, r_{el}^2} \tag{13.6}$$

$$R_B = \frac{1}{\sigma_B}\frac{r_{el}}{2r_{el}h_{el}}$$

$$R_{int} = \frac{1}{\sigma_B}\frac{g_{el}}{2r_{el}h_{el}}$$

where, σ_B is the electrolytic conductivity, h_{el} is the thickness of the gel electrolyte, and g_{el} is the gap between the electrodes. Using the interface resistance R_{int} as the resistance between the electrolyte-electrode pairs. The Kirchhoff's voltage and current equations for connections between the pairs are given by,

$$\begin{aligned} i_{CN,(i,j)} &= i_{pN,(i,j)} = -i_{pS,(i,j+1)} \\ i_{CE,(i,j)} &= i_{pE,(i,j)} = -i_{pW,(i+1,j)} \\ \phi_{pN,(i,j)} - \phi_{pS,(i,j+1)} &= R_{int}\, i_{CN,(i,j)} \\ \phi_{PE,(i,j)} - \phi_{PW,(i+1,j)} &= R_{int}\, i_{CE,(i,j)} \end{aligned} \tag{13.7}$$

where, $i_{CN,(i,j)}$ and $i_{CE,(i,j)}$ are the currents at the interface resistor (defined in the north and east directions), $i_{pN,(i,j)}$, $i_{pS,(i,j+1)}$, $i_{pE,(i,j)}$, $i_{pW,(i+1,j)}$ are the horizontal currents at the electrode-electrolyte pairs. The directionality of the interface currents and the horizontal currents from each electrode-electrolyte pair is considered to formulate the equations. In addition to the equations given by (13.7), as the boundary conditions, we assume that for all the electrolyte-electrode pairs at the boundaries, the directional horizontal edge potentials going outside the array are connected to the ground (set potential as 0) via the interface resistance.

The equations $(13.1 - 13.7)$ together with appropriate boundary conditions using Kirchhoff's current and voltage laws describe the set of governing equations that can predict the electrolytic current and potentials on the application of electrode potentials over the electrode array. Additionally, to define a switching potential operation on the electrode between two voltage steps, for the numerical stability of the formulation, we used a smoothed step function as a voltage-switching function $f_s(V_N, V_O)$ given by,

$$f_s(V_N, V_O) = V_O + (V_N - V_O)\left(\frac{1}{2} + \frac{1}{\pi}tan^{-1}\left(\frac{t - t_0}{\lambda}\right)\right) \tag{13.8}$$

where, V_O and V_N are the initial and the final voltages, t_0 is the offset time, and λ defines the sharpness of the voltage switching. An example of the voltage switching function from 0V to 1.5V with an offset time of 0.5s at various λ is shown in **Figure S18**.

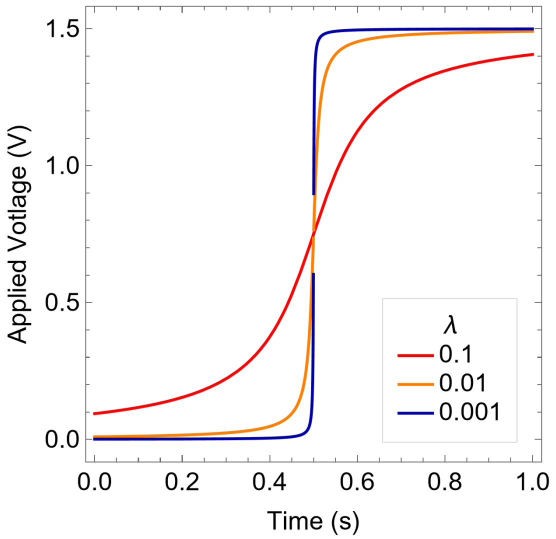

Figure S18. Voltage switching function for switching between two potentials. The figure shows voltage switching from 0V to 1.5V with offset time 0.5s at different values of λ.

Equations $(13.1 - 13.8)$ define a complete set of equations which can be solved together to calculate the time-dependent profile of currents, and electrolytic potentials over the interconnected grid of electrodes. As an example, we ran a simulation over a 7×7 electrode array with five activated electrodes with applied potentials ±2.5V using a step-up function and the rest of the electrodes with potential set to 0 V. We chose offset time 0.5 s, $\lambda = 10^{-4}$, initial voltage 0V and switching time 2.5s. The parameter relevant to Faradaic current such as exchange current density estimated from homogenous rate constant is assumed for simplicity and does not describe quinone electrochemical kinetics. **Figure S19(A)** and **(B)** shows applied potential and current distribution in two different cases plotted at 4s, (A) central electrode positive and neighboring electrodes negative voltages, (B) central electrode negative and neighboring electrodes positive voltages. The applied voltage, electrolytic current, and interfacial currents are plotted. The parameters used in the simulation are given in Table S4. All the simulations were performed using Mathematica 13 and the coupled algebraic and differential equations were solved using the *NDSolve* function. The Mathematica Notebook for the electrochemical network model used is available online at https://github.com/croningp/HybridComputation.

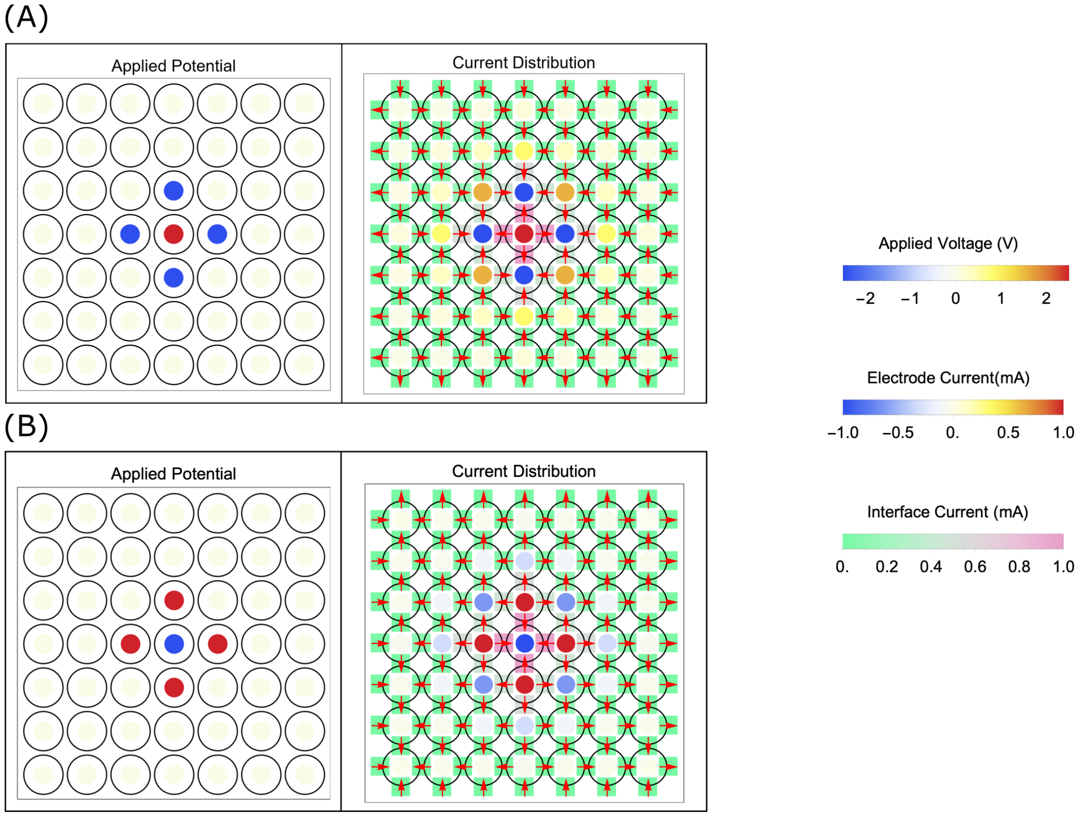

Figure S19. Application of potentials on the electrode array. (A) and (B) shows application of potentials on 7×7 electrode array. In the first case (A), the central electrode potential is +2.5 V and the potentials on four neighboring electrodes are –2.5V. In the second case (B), the central electrode potential is –2.5V and the potentials on four neighboring electrodes are +2.5V. Both potentials and currents are plotted at 4s.

13.2.2 pH changes over a single electrode

The generalized governing equations of the electrochemical network are composed of electrochemical kinetics defined by the Secondary Current Distribution model and the network equations using Kirchhoff's Current and Voltage laws. Considering that the Faradaic reactions occurring on the electrode create a positive or negative flux of H^+ ions which depend on the current flow defined by Butler-Volmer equations as a function of the applied voltages. We simplify our formulation by decoupling the electrochemical kinetics and buffer dynamics and also assuming that the electrochemical kinetics is independent of the local pH at the vicinity of the electrode. To predict the pH changes over the electrode, the electrochemical kinetics equation and localized buffer kinetics equations are solved together with time stepping. Additionally, we also assume that the localized pH changes are governed by equilibrium buffer kinetics only and are homogeneous in the vicinity of the individual electrodes such that pH changes can be applied homogeneously over the finite volume. Over a grid of electrodes, the homogenous region in the vicinity of the electrodes is defined by the cylindrical region with volume $V = \pi\,(r_{el} + g_{el}/2)^2 h_{el}$.

Considering the electrochemical kinetics involving electrochemical reactions where the electron transfer lead to the formation of H^+ and OH^- ions at the electrode surface. This could lead to local pH changes on the application of electrode potential, which is used as an analog chemical state for computational operations as described in the previous subsection. Here, we combine the electrochemical kinetics together with phosphate buffer dynamics to simulate the localized pH changes over the electrode array. The phosphate buffer equilibrium comprises the following equations, with $pk_1 = 12.35$, $pk_2 = 7.2$, $pk_3 = 2.15$.

$$k_1 = \frac{[\mathrm{H}^+][\mathrm{PO}_4^{-3}]}{[\mathrm{HPO}_4^{-2}]} \tag{13.9}$$

$$k_2 = \frac{[\mathrm{H}^+][\mathrm{HPO}_4^{-2}]}{[\mathrm{H}_2\mathrm{PO}_4^{-}]}$$

$$k_3 = \frac{[\mathrm{H}^+][\mathrm{H}_2\mathrm{PO}_4^{-}]}{[\mathrm{H}_3\mathrm{PO}_4]}$$

With total phosphate concentration is given by P_T considered as constant locally and hence $P_T = [\mathrm{PO}_4^{-3}] + [\mathrm{HPO}_4^{-2}] + [\mathrm{H}_2\mathrm{PO}_4^{-}] + [\mathrm{H}_3\mathrm{PO}_4]$. Therefore, for a given pH or $[\mathrm{H}^+]$ concentration, the concentrations of various phosphate species ($[\mathrm{PO}_4^{-3}]$, $[\mathrm{HPO}_4^{-2}]$, $[\mathrm{H}_2\mathrm{PO}_4^{-}]$, $[\mathrm{H}_3\mathrm{PO}_4]$) in the local volume are given by

$$[\mathrm{PO}_4^{-3}] = P_T\,\frac{k_1k_2k_3}{[\mathrm{H}^+]^3 + [\mathrm{H}^+]^2k_3 + [\mathrm{H}^+]k_2k_3 + k_1k_2k_3} \tag{13.10}$$

$$[\mathrm{HPO}_4^{-2}] = P_T\,\frac{[\mathrm{H}^+]k_2k_3}{[\mathrm{H}^+]^3 + [\mathrm{H}^+]^2k_3 + [\mathrm{H}^+]k_2k_3 + k_1k_2k_3}$$

$$[\mathrm{H}_2\mathrm{PO}_4^{-}] = P_T\,\frac{[\mathrm{H}^+]^2k_3}{[\mathrm{H}^+]^3 + [\mathrm{H}^+]^2k_3 + [\mathrm{H}^+]k_2k_3 + k_1k_2k_3}$$

$$[\mathrm{H}_3\mathrm{PO}_4] = P_T\,\frac{[\mathrm{H}^+]^3}{[\mathrm{H}^+]^3 + [\mathrm{H}^+]^2k_3 + [\mathrm{H}^+]k_2k_3 + k_1k_2k_3}$$

Here, we assume that the positive current produces $[\mathrm{H}^+]$ and the negative current consumes $[\mathrm{H}^+]$. In Δt time on the application of electrode potential, the total number of moles of $[\mathrm{H}^+]$ produced and consumed is given by Faraday's law as $m[\mathrm{H}^+]_n = \frac{i_{el}\Delta t}{n\,F}$ where i_{el} is the electrolytic current, n is the number of electrons transferred (n was assumed as 1 for simplicity), and F is Faraday's constant. Over the homogeneous volume, we can define the change in the moles of $[\mathrm{PO}_4^{-3}]$,

$[HPO_4^{-2}]$, $[H_2PO_4^-]$, $[H_3PO_4]$ are given by $(V\,[PO_4^{-3}] - \alpha\, m[H^+]_n)$, $V\,[HPO_4^{-2}] + (\alpha - \beta)\ m[H^+]_n$, $V\,[H_2PO_4^-] + (\beta - \gamma)\ [H^+]_n$, $V\,[H_3PO_4] + \gamma[H^+]_n$ together with the constraint $\alpha + \beta + \gamma = 1$. Using equations 13.9,

$$\frac{k_1}{[H^+]} = \frac{(V[PO_4^{-3}] - \alpha\, m[H^+]_n)}{V[HPO_4^{-2}] + (\alpha - \beta)m[H^+]_n} \tag{13.11}$$

$$\frac{k_2}{[H^+]} = \frac{(V[HPO_4^{-2}] + (\alpha - \beta)m[H^+]_n)}{V[H_2PO_4^-] + (\beta - \gamma)m[H^+]_n}$$

$$\frac{k_3}{[H^+]} = \frac{(V[H_2PO_4^-] + (\beta - \gamma)m[H^+]_n)}{V[H_3PO_4] + \gamma m[H^+]_n}$$

where, $m[H^+]_n$ are the moles of new $[H^+]$ added or consumed, $[H^+]$ is the final concentration required for estimating pH. This can be simplified by describing the concentration change from $m[H^+]_n$ as $[H^+]_n = m[H^+]_n/V$. Hence, equations 13.11 become,

$$k_1 = \frac{[H^+]([PO_4^{-3}] - \alpha[H^+]_n)}{[HPO_4^{-2}] + (\alpha - \beta)[H^+]_n} \tag{13.12}$$

$$k_2 = \frac{[H^+]([HPO_4^{-2}] + (\alpha - \beta)[H^+]_n)}{[H_2PO_4^-] + (\beta - \gamma)[H^+]_n}$$

$$k_3 = \frac{[H^+]([H_2PO_4^-] + (\beta - \gamma)[H^+]_n)}{[H_3PO_4] + \gamma[H^+]_n}$$

with $[H^+]_n = \frac{1}{n\,V\,F} i_{el}\Delta t$ and $\alpha + \beta + \gamma = 1$. Please note that this is an over simplified solution to predict the pH changes addition or consumption of $[H^+]$ in a system, considering a strong acid or base as the cause of pH change. Also, this assumes that the electroneutrality assumption is valid throughout the system due to the presence of supporting electrolyte in high concentration and water equilibrium is ignored. As an example, assuming the buffer kinetics is fast and homogeneous over the hemispherical volume $V = \frac{2}{3}\pi\, r_e^3$ (with radius $r_e = 0.5$mm), we tested the effect of applied current on pH on a single electrode at different ranges of positive and negative currents. The positive current generates the positive flux of $[H^+]$ which decreases the pH and negative current increases the pH. The starting pH was assumed to be 7 and the total phosphate concentration was assumed to be 20 mM.

The changes in the pH over a single electrode on the application of current are shown in **Figure S20**.

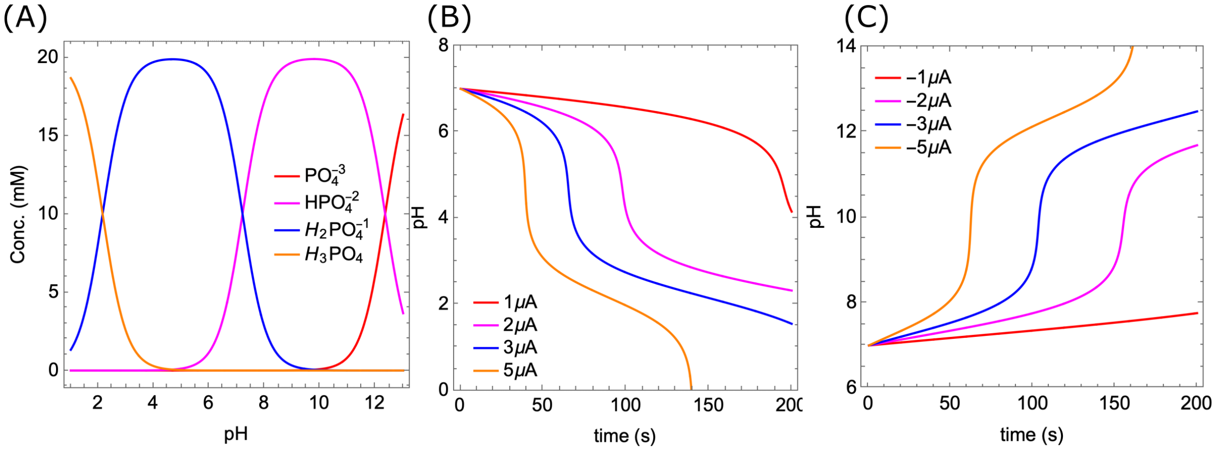

Figure S20. Application of current on single electrode. (A) Distribution of the concentration of phosphate species as a function of pH (total phosphate concentration 20mM). (B) pH within the homogeneous volume *vs*. time on the application of positive current in the range 1 μA–5 μA. (C) pH within the homogeneous volume *vs*. time on the application of negative current in the range 1 μA–5 μA.

13.2.3 pH changes over a grid of electrodes

To extend buffer dynamics formulation over the electrode array, we combined governing equations of the electrochemical network together with the current-induced pH change to create a generalized model to predict the distribution of pH over the electrode array. By combining the electrochemical network model with localized buffer dynamics, we can predict time-dependent currents, and pH distribution over the electrode array. To achieve this, first, the electrochemical network model is solved and time-dependent electrolytic current profiles for each electrode were calculated. Using the electrochemical current profile, pH changes over each electrode were numerically calculated with a time step of 0.1s. As an example, we considered a 6×6 electrode array and applied random potentials on the electrodes in the range (–1, +1) for 10 potential switching steps. The offset time was set to 0.5s, $\lambda = 10^{-4}$, switching time 2.5s, and initial voltage on all the electrodes as 0.25V. All the parameters used in the simulation are given in **Table S4**. A snapshot output of the simulation is shown in **Figure S21**, showing applied potential, current distribution over the electrode array (electrode current (vertical) and interfacial current (horizontal)) and local pH distribution over the electrode arrays.

Table S4. List of parameters used in the electrochemical network simulations.

	Parameter	Value	Comments
**1**	Electrode Radius r_{el}	$1\ mm$	Assumed
**2**	Electrode Gap g_{el}	$1\ mm$	Assumed
**3**	Height h_{el}	$1\ mm$	Assumed
**4**	Double Layer Capacitance	$0.2\ F/m^2$	Assumed
**5**	Concentration background electrolyte	$100\ mM$	For improving solution conductivity
**6**	Concentration of electroactive species	$100\ mM$	Assumed
**7**	Diffusion Constant (background)	$1 \times 10^{-9} m^2/s$	Characteristic value
**8**	Diffusion Constant (electroactive)	$1 \times 10^{-9} m^2/s$	Characteristic value
**9**	Homogeneous Rate Constant	$2.5 \times 10^{-6} m/s$	Assumed
**10**	Valence	1	Assumed
**11**	Bulk Conductivity	$0.75\ m^{-1}\Omega^{-1}$	Calculated
**16**	Exchange Current Density	$24.12\ A/m^2$	Calculated
**17**	Total Phosphate Concentration	$20 \times 10^{-3} M$	Assumed
**18**	Equilibrium potential (E_0)	0	Assumed
**19**	Charge Transfer Coefficient (Anodic and Cathodic)	0.5	α_a, α_c

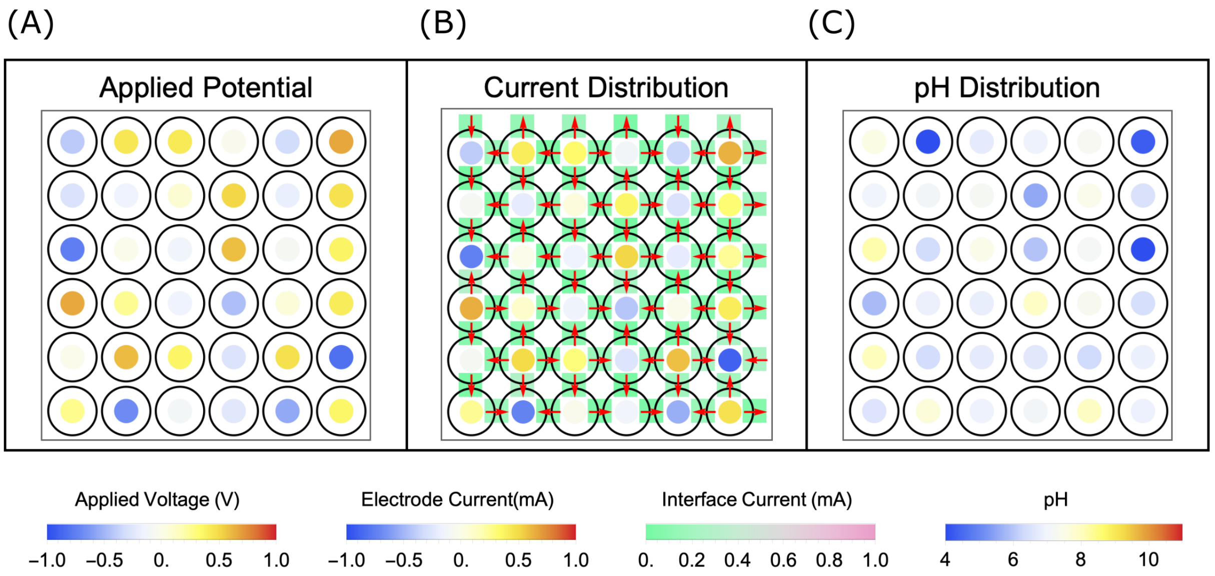

Figure S21. Visualization of the output of the electrochemical network model coupled with localized buffer dynamics. The figure shows applied potential, electrode current, interfacial currents, and observed pH changes over the electrode array on application of random potentials. The pH distribution ranges from blue (pH=4) to red (pH=11) with pH range 4–11.

13.2.4 Programmable pH changes over electrode arrays using a feedback loop.

As an extension of the programmable pH changes over the electrode array, we implemented a feedback loop based on local pH readout to program or control the pH changes and sustain a target pH on a set of given electrodes. Inspired by the experimental design, we introduce two types of electrodes, **ACTIVE** and **INACTIVE**. The active electrodes consist of working electrodes which are used for computational operations and as well as the counter electrodes to support the Faradaic reactions and current flow on the working electrodes. The current on **ACTIVE** electrodes (including both working and counter electrodes) is dynamic and depends on the applied voltages. The inactive electrodes represent the electrodes over the array which are not part of the computation process and stay idle over the array. For simplicity, all the inactive electrodes were set at very small constant potential $V_{app} \sim 0V$. Additionally, if necessary, an additional constraint can be added by setting a zero current condition on the inactive electrodes. This constraint can be made more realistic by setting a very small constant current ($1pA$) as fixed leakage current on inactive electrodes which could be an outcome of electronics design (CMOS electronics). Adding a constant current condition instead of Butler Volmer kinetics makes it independent of applied voltage on inactive electrodes (implemented in a closed-loop system for solving combinatorial optimization problems). To implement the feedback loop, for simplicity, we used proportional logic (which in principle can be extended to full Proportional Integral Derivative as implemented in experimental control) where the magnitude of the applied voltage on the working electrode is proportional to the pH difference between the current pH (pH_C) and the target pH (pH_T), which is given by,

$$V_{app,WE} = -\alpha(pH_T - pH_c) \tag{13.13}$$

where α is the proportionality coefficient. In the simulations, the voltage on the counter electrode is estimated by the mean value of required voltages on the surrounding working electrodes. Hence, the applied voltage on the counter electrode is given by,

$$V_{app,CE} = \frac{1}{N}\sum_{i=1}^{N} V_{WE,i} \tag{13.14}$$

where N is the neighboring working electrodes (in our case maximum $N = 4$), $V_{WE,i}$ is the applied potential on the ith neighboring working electrode. So, for each working electrode, there needs to be at least a single counter electrode as its nearest neighbor such that stable pH changes can be created without influencing the other working electrodes. The pH changes on the counter electrodes are not important as they do not represent computational states and are ignored for computation. Once the voltages on working and counter electrodes are estimated, the voltages are applied to all the active electrodes using the voltage switching function given in equation 13.8. As an example, to test the feedback control of pH over a single electrode, we performed a parametric study to sustain two different pH values 4 and 10. So, over a grid of 5×5 electrodes, we set the central electrode as a single working electrode surrounded by four counter electrodes. The offset

time was set to 0.25s, $\lambda = 10^{-4}$ and initial voltage on all the electrodes as 1×10^{-6} V. We vary the proportionality coefficient (α) and stepping time for the potential shift (t_S) to test the algorithm's ability to control the pH changes. **Figure S22**(A) and (B) show a time-dependent profile of pH over the working electrode with pH $= 4$ as the target pH and stepping time as 1.0 and 2.5 seconds. Similarly, **Figure S22**(C) and (D) show a time-dependent profile of pH over the working electrode with pH $= 10$ as the target pH and stepping time as 1.0 and 2.5 seconds. In both cases, we used pH $= 7$ as the starting pH and observed stronger oscillations at the target pH with increases in α and t_S. At lower α values, the fluctuations in pH after achieving the target pH are much lower or almost constant, however, the time to achieve the target pH is much higher. At higher α values, the system achieves target pH much faster however oscillates around the target value. So, there is a trade-off between the accuracy and time required for achieving target pH over the electrode array, which defines the selection of α and t_S for a given computational problem. However, it is important to note that physical parameters such as range of applied voltage, buffer concentration, geometry of the electrode arrays all play a crucial role in the time-dependent pH dynamics to achieve a target pH.

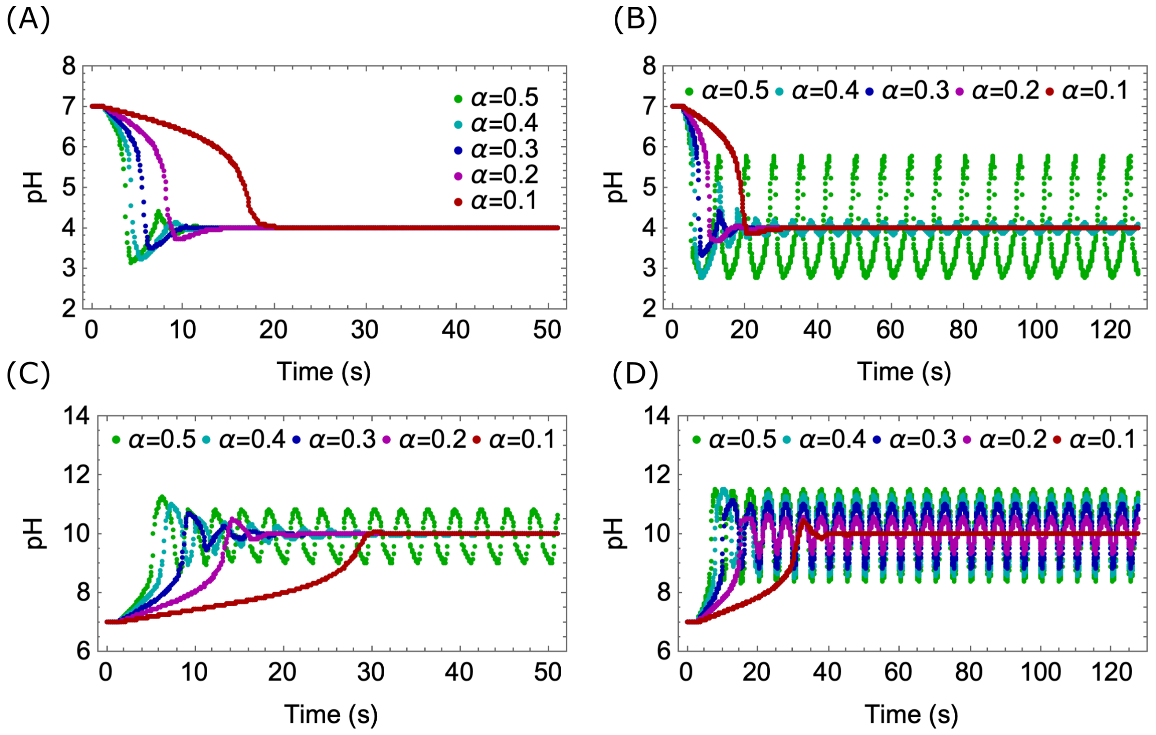

Figure S22. Programmable pH changes on a single electrode. (A) and (B) shows time-dependent pH profiles with pH $= 4$ as the target pH at different α values with t_S as 1s and 2.5s. Similarly, (C) and (D) show pH profiles with similar parameters for target pH $= 10$.

Using a similar approach, we extended our formulation to program pHs over multiple working electrodes. As the basic examples, we used two different examples with four working electrodes over a 5×5 electrode array as shown in **Figure S23**. With the four working electrodes in the

configuration shown in the figure, there are a total of 12 counter electrodes with four counter electrodes surrounding each working electrode. The general list of experimental factors used in the electrochemical network and buffer dynamics simulations are the same as described in **Table S4** and additional parameters are given in **Table S5**.

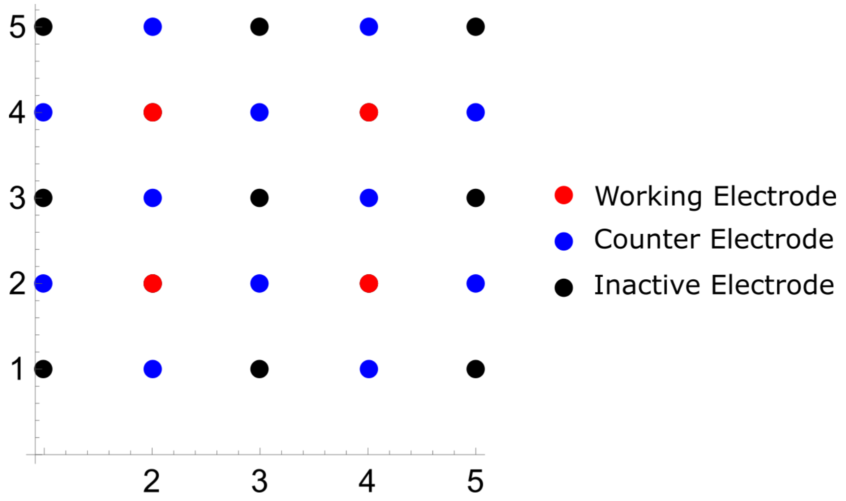

Figure S23. Four working electrodes configuration over the electrode array. Working, Counter and Inactive electrodes are shown in red, blue, and black respectively.

Table S5. List of additional parameters used in the electrochemical network simulations.

	**Parameter**	**Value**	**Comments**
**1**	Offset Time	$0.25s$	Electrode potential offset time for shifting
**2**	λ	1×10^{-4}	Parameter describing sharpness of potential shift
**3**	V_{init}	1×10^{-6}	Initial potential on all electrodes
**4**	t_{step}	$0.1s$	Time stepping for pH calculations

To test the system's capability to achieve multiple pHs over the electrode array simultaneously, we selected two different goals. In the first case we set as target two electrodes at pH $= 4$ and the other two electrodes at pH $= 8$, to achieve this we set $\alpha = 0.1$ and $t_S = 1$s and ran the simulation up to 250 steps. In the second case, we set as target different pHs on all four electrodes as pH $= 4, 6, 9, and\ 11$ respectively. In this case, to achieve a quicker response, we set $\alpha = 0.25$ and $t_S = 3$s and ran the simulation up to 50 steps. In both cases starting with pH $= 7$ on all the electrodes and similar other parameters, we were able to achieve all the target pHs, as shown in **Figure S24**(A) and (C). The applied potentials on all the working electrodes for the two targets are shown in **Figure S24** (B) and (D). These examples prove the capability of the feedback control loop system to achieve target pHs which can be utilized for solving computation problems. The Mathematica Notebook for the feedback loop is available at: https://github.com/croningp/HybridComputation

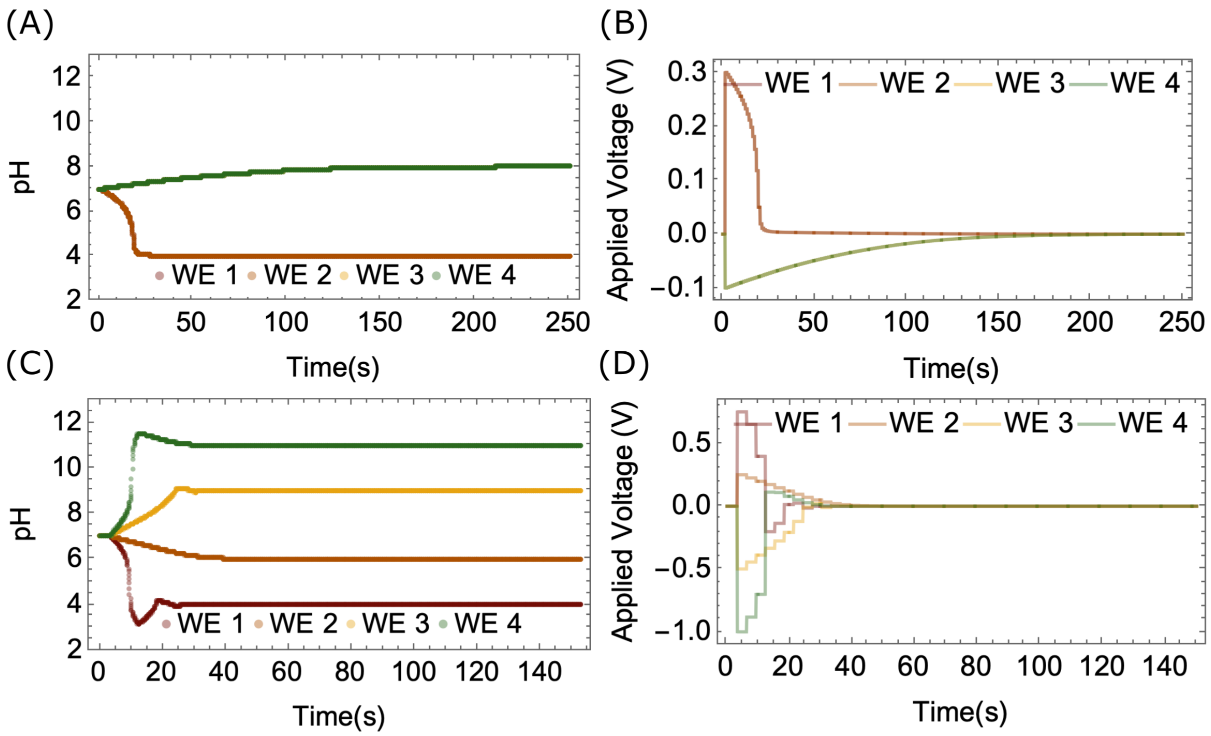

Figure S24. Simultaneous pH control on four working electrodes. (A) and (B) shows time-dependent pH and applied potential on the working electrodes with the target to achieve pH $= 4$ and pH $= 8$ over a pair of two electrodes (C) and (D) shows the same with target pHs $= 4, 6, 9,$ and 11.

13.3 Dynamic pH control towards solving optimization problems

In this section, we expand our multielectrode pH control logic to dynamic multielectrode logic based on time-dependent abstract states, similar to the ones used in the solution to combinatorial optimization problems using a stochastic gradient descent algorithm. In the experiments, we combine a feedback control loop to program pH over an array of electrodes together with the stochastic gradient descent method to solve quadratic combinational optimization problems. In this section, we will explore the features of dynamic pH control where abstract mathematical states are mapped to pH values. We use abstract states similar to the Ising/QUBO formulation solving solving combinatorial optimization problems. The Ising spin variables are defined by s_i= (–1, +1) and the Quantum Unconstrained Binary Optimization (QUBO) abstract states can be defined by the transformation $s_i \rightarrow -1 + 2x_i$, such that Ising spin -1 corresponds to QUBO variable 0 and +1 corresponds to variable +1. We define a linear transformation between Ising/QUBO variables and pH, such that within the dynamic feedback loop, the abstract variables and pH can be used interchangeably.

As a simple example of Ising/QUBO formulation, the number partition problem is defined as: for a given set of positive numbers $S = \{n_1, n_2, n_3 \ldots n_N\}$, find the two disjoint sets Q and $S -$

Q such that the sum of all the elements in both sets is equal if exists. This problem can be mapped directly to an Ising formulation whose Hamiltonian is given by,[13]

$$H = A\left(\sum_{i=1}^{N} n_i s_i\right)^2 \tag{13.15}$$

As an example, consider a number set $S = \{1, 2, 4, 7\}$. Here, the Ising Hamiltonian for the Number Partitioning Problem was created and translated into the Quantum Unconstrained Binary Optimization (QUBO) formulation with the transformation $s_i \rightarrow -1 + 2\mathrm{x}_i$ such that Ising spin -1 corresponds to QUBO variable 0 and +1 corresponds to variable 1. The Hamiltonian after transformation from Ising to QUBO with four variables $(\mathrm{x}_1, \mathrm{x}_2, \mathrm{x}_3, \mathrm{x}_4)$ is given by,

$$\begin{aligned} H_{\mathrm{QUBO}} = 196 - 56\mathrm{x}_1 + 4{\mathrm{x}_1}^2 - 112\mathrm{x}_2 + 16\mathrm{x}_1\mathrm{x}_2 + 16{\mathrm{x}_2}^2 - 224\mathrm{x}_3 + 32\mathrm{x}_1\mathrm{x}_3 \\ + 64\mathrm{x}_2\mathrm{x}_3 + 64{\mathrm{x}_3}^2 - 392\mathrm{x}_4 + 56\mathrm{x}_1\mathrm{x}_4 + 112\mathrm{x}_2\mathrm{x}_4 + 224\mathrm{x}_3\mathrm{x}_4 \\ + 196{\mathrm{x}_4}^2 \end{aligned} \tag{13.16}$$

Instead of solving the number partition problem by stochastic gradient descent method as already been demonstrated in the experiments, here we explore the features of dynamic pH control logic such as pH convergence, dynamically applied potentials on the working and counter electrodes and electrolytic currents. Inspired by the QUBO variables, we selected four abstract states and explore the pH evolution based on random exploration. The evolution of the abstract states was based on adding random noise sampled from a normal distribution such that $\mathrm{x_i}(t+1) = \mathrm{x_i}(t) + \eta(t)$, where $\eta(t)$ is the noise term. If the abstract state reaches the boundary, a hard condition was applied to prevent overshooting as if $\mathrm{x_i}(t) > 1, \mathrm{x_i}(t) = 1$ & if $\mathrm{x_i}(t) < 0, \mathrm{x_i}(t) = 0$. The four abstract states were mapped to four working electrodes. The working electrodes and the surrounding counter electrodes for four variables are shown in **Figure S25**. We selected two different mappings between the abstract states and pH; in the first case, abstract states 0 and 1 correspond to pH states 4 and 11, and in the second case, the abstract states 0 and 1 correspond to pH states 5 and 8. The list of physical parameters used in the simulations is given in Table S6. The list of additional parameters for dynamic pH control used in the simulation is given in Table S7.
In the simulations, the applied or target pHs were estimated purely based on the abstract variables and a fixed time was set for the pH to attain the target value. The new abstract states were not chosen from the final pH but directly from the previous abstract states from the algorithm. However, instead of setting a fixed time to achieve a target pH, dynamic time can be set by introducing an additional logic setting a threshold range where the pH is considered to be achieved to the target. In that case, the abstract states corresponding to the emerging pHs can be used to update the new abstract states. The main aim of the simulation engine is to observe features of dynamic pH control over the electrode array similar to the experimental control.

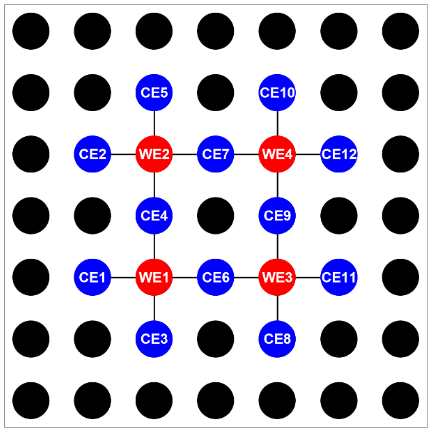

Figure S25. Mapping of 4 abstract variables on the electrode array. The figure shows the electrode array and mapping 4 variables on working electrodes. For each variable, a working electrode is shown in red. The surrounding counter electrodes are shown in blue and the rest of the electrodes in black are inactive.

Table S6. List of parameters used in electrochemical network simulations.

	**Parameter**	**Value**	**Comments**
**1**	Electrode Radius r_{el}	$0.5\ mm$	Assumed
**2**	Electrode Gap g_{el}	$1\ mm$	Assumed
**3**	Height h_{el}	$1\ mm$	Assumed
**4**	Double Layer Capacitance	$0.2\ F/m^2$	Assumed
**5**	Concentration background electrolyte	$100\ mM$	For improving solution conductivity
**6**	Concentration of electroactive species	$100\ mM$	Experimental Design
**7**	Diffusion Constant (background)	$1 \times 10^{-9} m^2/s$	Characteristic value
**8**	Diffusion Constant (electroactive)	$1 \times 10^{-9} m^2/s$	Characteristic value
**9**	Homogeneous Rate Constant	$2.5 \times 10^{-6} m/s$	Assumed
**10**	Valence (background electrolyte)	1	Assumed
**11**	Bulk Conductivity	$0.75\ m^{-1}\Omega^{-1}$	Calculated
**12**	Exchange Current Density	$24.12\ A/m^2$	Calculated
**13**	Total Phosphate Concentration	$20 \times 10^{-3} M$	Assumed
**14**	Equilibrium potential (E_0)	0.0	Assumed
**15**	Charge Transfer Coefficient (Anodic and Cathodic)	0.5	α_a, α_c
**16**	Number of electrons transfer (n)	1	Assumed for electrochemical reaction

Table S7. List of additional parameters used in the electrochemical network simulations.

	Parameter	Value	Comments
**1**	Offset Time	$0.25s$	Electrode potential offset time for shifting
**2**	λ	1×10^{-4}	Parameter describing sharpness of potential shift
**3**	t_S	$4.0s$	Time step for a potential shift in seconds
**4**	i_l	$1\,pA$	Leakage current on inactive electrodes
**5**	V_{init}	1×10^{-6}	Initial potential on all electrodes
**6**	t_{step}	$0.1s$	Time stepping for pH calculations
**7**	t_{switch}	$60.0s$	Switch time between two pH states on the working electrode (independent of the convergence)
**8**	α	0.1	Proportional Factor for applied voltage based on pH difference
**9**	μ	0.0	Mean value of Normal distribution for adding noise
**10**	σ_{NP}	0.1	Standard deviation for the Normal distribution for adding noise

The features of dynamic pH control in the stochastic evolution of abstract variables and pHs are shown in **Figure S26** and **Figure S27**. **Figure S26** shows the stochastic evolution of abstract variables with variable 0 mapping to pH 4 and variable 1 mapping to pH 11. Similarly, **Figure S27** shows the stochastic evolution of abstract variables with variable 0 mapping to pH 5 and variable 1 mapping to pH 8. In both cases, extremely stable pHs at different target values are obtained due to low α values. As shown previously, with the increase in α the oscillatory behavior becomes more prominent. See https://github.com/croningp/HybridComputation for details and examples in Mathematica Notebooks.

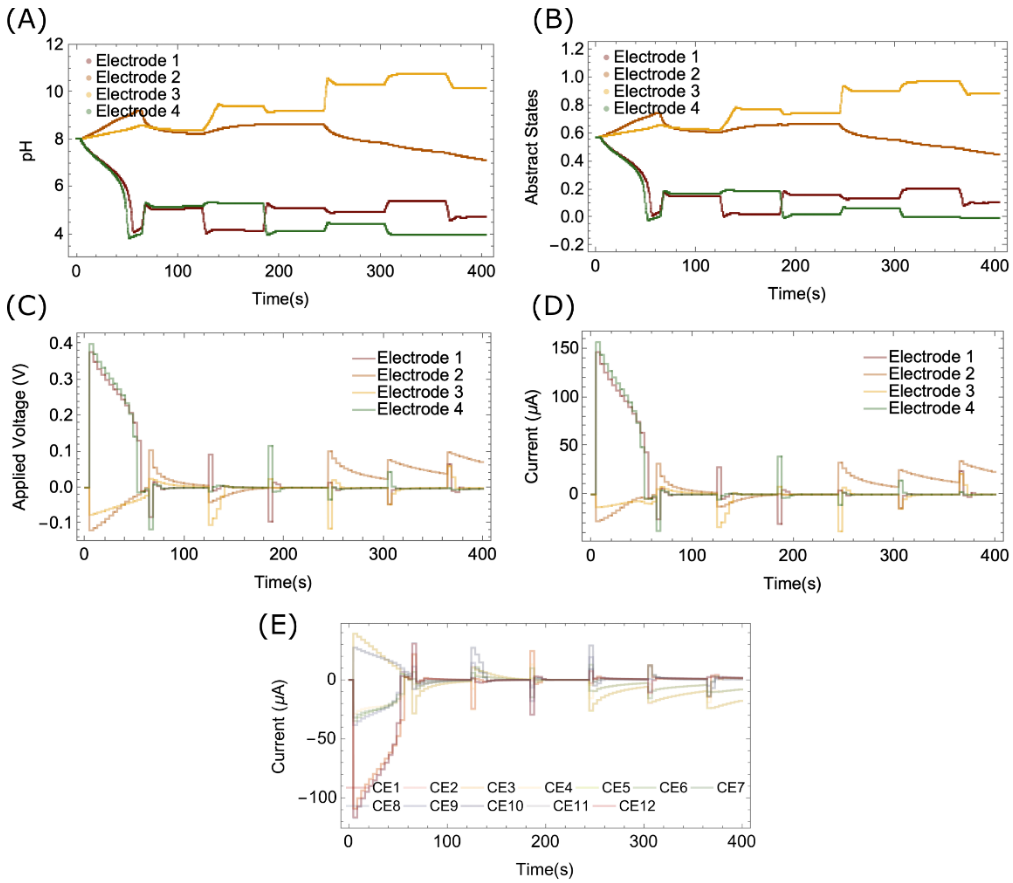

Figure S26. Stochastic evolution of pH based on abstract states (0: pH 4, 1: pH 11). (A) pH on the electrode arrays over the computation time (starting pH was set to 8), (B) Abstract states estimated from the observed pH over the computation time, (C) Applied voltage on the electrodes during the feedback control loop, (D) Current flowing through the electrode on the application of the voltage leading to pH changes, (E) Current flowing through the surrounding counter electrodes.

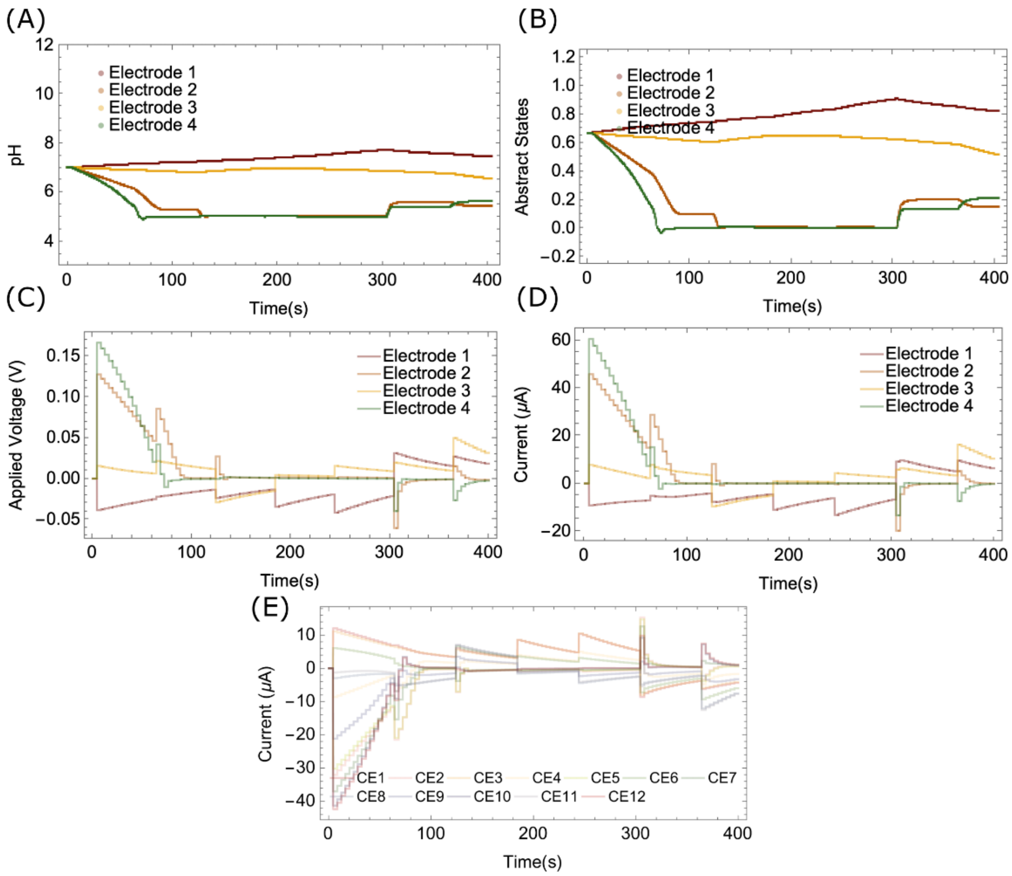

Figure S27. Stochastic evolution of pH based on abstract states (0: pH 5, 1: pH 8). (A) pH on the electrode arrays over the computation time (starting pH was set to 7), (B) Abstract states estimated from the observed pH over the computation time, (C) Applied voltage on the electrodes during the feedback control loop, (D) Current flowing through the electrode on the application of the voltage leading to pH changes, (E) Current flowing through the surrounding counter electrodes.

References

(1) Horsman, C.; Stepney, S.; Wagner, R. C.; Kendon, V. When Does a Physical System Compute? *Proc. R. Soc. A.* **2014**, *470* (2169), 20140182. https://doi.org/10.1098/rspa.2014.0182.
(2) McMillan, P. F.; Clary, D. C.; Wales, D. J. The Energy Landscape as a Unifying Theme in Molecular Science. *Philosophical Transactions of the Royal Society A: Mathematical, Physical and Engineering Sciences* **2004**, *363* (1827), 357–377. https://doi.org/10.1098/rsta.2004.1497.
(3) Wales, D. *Energy Landscapes: Applications to Clusters, Biomolecules and Glasses*; Cambridge University Press, 2003.
(4) Bennett, C. H. The Thermodynamics of Computation—a Review. *Int J Theor Phys* **1982**, *21* (12), 905–940. https://doi.org/10.1007/BF02084158.
(5) Guo, S. Y.; Friederich, P.; Cao, Y.; Wu, T. C.; Forman, C. J.; Mendoza, D.; Degroote, M.; Cavell, A.; Krasecki, V.; Hickman, R. J.; Sharma, A.; Cronin, L.; Gianneschi, N.; Goldsmith, R. H.; Aspuru-Guzik, A. A Molecular Computing Approach to Solving Optimization

Problems via Programmable Microdroplet Arrays. *Matter* **2021**, *4* (4), 1107–1124. https://doi.org/10.1016/j.matt.2021.03.002.
(6) Dueñas-Díez, M.; Pérez-Mercader, J. How Chemistry Computes: Language Recognition by Non-Biochemical Chemical Automata. From Finite Automata to Turing Machines. *iScience* **2019**, *19*, 514–526. https://doi.org/10.1016/j.isci.2019.08.007.
(7) Lucas, A. Ising Formulations of Many NP Problems. *Frontiers in Physics* **2014**, *2*.
(8) Hayes, B. Computing Science: Can't Get No Satisfaction. *American Scientist* **1997**, *85* (2), 108–112.
(9) Jiang, S.; Britt, K. A.; McCaskey, A. J.; Humble, T. S.; Kais, S. Quantum Annealing for Prime Factorization. *Sci Rep* **2018**, *8* (1), 17667. https://doi.org/10.1038/s41598-018-36058-z.
(10) Dattani, N. S.; Bryans, N. Quantum Factorization of 56153 with Only 4 Qubits. arXiv November 27, 2014. https://doi.org/10.48550/arXiv.1411.6758.
(11) Anschuetz, E.; Olson, J.; Aspuru-Guzik, A.; Cao, Y. Variational Quantum Factoring. In *Quantum Technology and Optimization Problems*; Feld, S., Linnhoff-Popien, C., Eds.; Lecture Notes in Computer Science; Springer International Publishing: Cham, 2019; pp 74–85. https://doi.org/10.1007/978-3-030-14082-3_7.
(12) Sharma, A.; Ng, M. T.-K.; Gutierrez, J. M. P.; Jiang, Y.; Cronin, L. A Probabilistic Chemical Programmable Computer. **2022**. https://doi.org/10.48550/ARXIV.2204.13493.
(13) Lucas, A. Ising Formulations of Many NP Problems. *Front. Physics* **2014**, *2*. https://doi.org/10.3389/fphy.2014.00005.
